# Protein intake from 0 to 18 years of age and its relation to health: a systematic literature review for the 5th Nordic Nutrition Recommendations

**DOI:** 10.3402/fnr.v57i0.21083

**Published:** 2013-05-23

**Authors:** Agneta Hörnell, Hanna Lagström, Britt Lande, Inga Thorsdottir

**Affiliations:** 1 Department of Food and Nutrition, Umeå University, Umeå, Sweden; 2 Turku Institute for Child and Youth Research, University of Turku, Turku, Finland; 3 Division of Public Health, Norwegian Directorate of Health, Oslo, Norway; 4 Unit for Nutrition Research, School of Health Sciences, University of Iceland and Landspitali National University Hospital, Reykjavik, Iceland

**Keywords:** BMI, bone health, growth, overweight, puberty

## Abstract

The present systematic literature review is a part of the 5th revision of the Nordic Nutrition Recommendations. The aim was to assess the health effects of different levels of protein intake in infancy and childhood in a Nordic setting. The initial literature search resulted in 435 abstracts, and 219 papers were identified as potentially relevant. Full paper selection resulted in 37 quality-assessed papers (4A, 30B, and 3C). A complementary search found four additional papers (all graded B). The evidence was classified as convincing, probable, limited-suggestive, and limited-inconclusive. Higher protein intake in infancy and early childhood is convincingly associated with increased growth and higher body mass index in childhood. The first 2 years of life is likely most sensitive to high protein intake. Protein intake between 15 E% and 20 E% in early childhood has been associated with an increased risk of being overweight later in life, but the exact level of protein intake above which there is an increased risk for being overweight later in life is yet to be established. Increased intake of animal protein in childhood is probably related to earlier puberty. There was limited-suggestive evidence that intake of animal protein, especially from dairy, has a stronger association with growth than vegetable protein. The evidence was limited-suggestive for a positive association between total protein intake and bone mineral content and/or other bone variables in childhood and adolescence. Regarding other outcomes, there were too few published studies to enable any conclusions. In conclusion, the intake of protein among children in the Nordic countries is high and may contribute to increased risk of later obesity. The upper level of a healthy intake is yet to be firmly established. In the meantime, we suggest a mean intake of 15 E% as an upper limit of recommended intake at 12 months, as a higher intake may contribute to increased risk for later obesity.

Both quality and quantity of protein intake in infancy and childhood are of interest with regard to later risk of non-communicable diseases (NCDs) (1). In the Nordic setting, the quantity of protein intake is of utmost importance, as its quality rarely is a problem. The hypothesis that high protein in infants’ diet stimulates growth and concurrently increases the likelihood of overweight and obesity later in life was first proposed in 1995 by Rolland-Cachera et al. (2). Several Nordic studies have since confirmed this association (3–5). The growth association has been thought to depend on the stimulating effect of high protein intake on insulin-like growth factor 1 (sIGF-I), which in turn may result in more rapid growth and increased muscle mass as well as fat mass (2, 6–9).

Rapid growth during the first year of life has been associated with an increased risk of overweight and obesity later in life in several epidemiological studies (7–9). Infants who are breastfed during the first months of life grow at a slower rate in infancy than those that are bottle-fed (10, 11). It has been postulated that part of the explanation is related to higher protein content of infant formula compared with mother's milk (12).

Other adverse health outcomes of high protein intake early in life have also been suggested. A systematic literature review (SLR) was needed to improve the knowledge about possible negative effects of a high protein intake. This is essential to enable formulating advice about appropriate foods to give infants and young children during the transition from breast milk to family foods, as well as for deciding safe levels for the composition of infant formulas and follow-on formulas. Further, it is important to explore the association between older children's protein intake and health.

According to the World Health Organization/Food and Agriculture Organization (WHO/FAO), reference values for protein intake is 0.9 g/kg/day from 3 to 18 years of age for boys and from 3 to 15 years of age for girls (24). Between 15 and 18 years of age, the level decreases slightly for girls to 0.8 g/kg/day. Combining this reference value with the reference values for energy intake for age and sex, this equates to about 5 protein energy percentage (PE%) at three years of age gradually increasing to about 7–9 PE% at 17 years of age for boys and girls, respectively. The intake of protein in the Nordic countries is, as in many industrialized countries, more than sufficient to meet physiological requirements among children (Table 1).

**Table 1:** Protein intake among children in the Nordic countries (percent of total energy, mean values given for boys/girls, or total)

	12 months	2 years	4 years	6 years	8 years	9 years	10 years	11 years	13 years
Denmark^1^–^2^		11.6/12.4			13.4/-				
Finland^3^–^4^	15/16	16/17	15/15	16/15			16.1/16.1		
Iceland^5^–^6^	15.6/14.8			15.7/15.0					
Norway^7^–^10^	13.1/13.0	14.6/14.9	14.2/14.1			14.5/14.0			14.8/14.2
Sweden^11^–^12^	12.7/12.9		14.4/14.4		15.4/15.4			15.9/15.4	

1 Children aged 2.5 years (13).

2 Boys aged 8 years (14).

3 Children aged 1–6 years were born in 2003 (12 months), 2001–2002 (2 years), 1999–2000 (4 years) (15).

4 Children aged 10 years (16).

5 Children aged 1 year (17).

6 Children aged 6 years (national data) (18).

7 Children aged 1 year, breastfed infants not included (national data) (19).

8 Children aged 2 years (national data) (20).

9 Children aged 4 years (national data) (21).

10 Children aged 9 and 13 years (national data) (22).

11 Children aged 1 year (4).

12 Children aged 4, 7, and 11 years (national data) (23).

In 2010, the Nordic Council of Ministers launched a project aimed at reviewing the scientific basis of the Nordic Nutrition Recommendations (NNR) issued in 2004 (25) and where necessary update a 5th edition. The NNR5 project is mainly focused on a revision of those areas in which new scientific knowledge with special relevance for the Nordic setting has emerged since the 4th edition. A number of systematic literature reviews (SLRs) will form the basis for the update.

The present SLR is focused on protein intake in infancy and childhood and the association with several different health outcomes.

## Aims

The overall aim was to review recent scientific data on the short- and long-term health effects of different levels of protein intake in infancy and childhood, in order to appraise the present recommendations in a Nordic setting.

### Research/key questions

1. What are the effects of different intakes and different sources of protein (animal- or plant-based) in infancy and childhood, while considering other energy-giving nutrients at the same time, on functional or clinical outcomes, including growth and development?
2. What are the effects of different intakes and different sources of protein (animal- or plant-based) in infancy and childhood, while considering other energy-giving nutrients at the same time, on well-established markers or indicators of functional or clinical outcomes, such as serum lipids, glucose and insulin, blood pressure, body weight, body composition, and bone mineral density, in childhood, adolescence, and adulthood?

Limits: Published since January 2000, human subjects. See below for inclusion and exclusion criteria and Appendix 1 for search terms.

## Methods

### Search terms

The main protein group in the NNR5-project managed the search and defined search terms in collaboration with the *Infant and Young Child* group and Hege Sletsjøe, librarian at the Norwegian Directorate of Health, Oslo, Norway. The search terms are presented in Appendix 1.

### Inclusion and exclusion criteria

The group focused on protein intake among healthy children. Inclusion criteria in the abstract screening process were the following: English or Nordic language, study population relevant to the Nordic countries.

Papers were excluded if they focused on premature or sick children, if the study population was deemed too different from a Nordic population, if intake data was not measured in childhood, if the outcome did not match the research questions, or if the paper was a general overview rather than an SLR.

### Search results

**Fig. 1:**
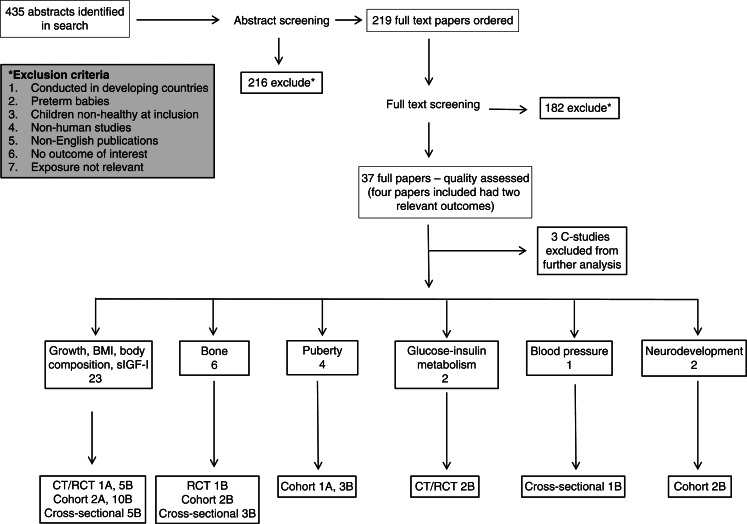
Overview search results of SLR on protein intake in childhood and health outcomes

The search was run in January 2011, including all relevant population groups and clinical outcomes. The main protein group did a first scan of the abstracts and sent all abstracts relevant for the age group 0–18 years (*n*=435) to our group (Fig. 1). Abstract screening was conducted in February–March 2011 according to the guide for conducting SLRs for the 5th edition of the NNR (26).

A total of 219 full papers were ordered, of which 182 papers were immediately excluded (most were excluded because they did not include the research questions or were general overviews rather than SLRs), leaving 37 papers selected for quality assessment (8 clinical trials, 19 cohort studies, and 10 cross-sectional studies). Reasons for exclusion are provided in Appendix 2.

A complementary search was performed in February 2012 covering the time since the first search until the end of December 2011. The abstracts were similarly evaluated for full paper reading. Included complementary papers were quality assessed and used to evaluate the conclusion of the SLR, as supporting or not.

### Quality assessment, grading, and reporting of evidence

The 37 included papers were quality assessed using the quality assessment tools (QAT) received from the NNR5 secretariat (26). These contained a number of questions regarding several methodological aspects of the studies, including questions about, for example, study design, population characteristics, exposure and outcome measures, dietary assessment, and confounders.

The quality assessment resulted in the following grading – clinical trials: 1A, 6B, 1C; prospective cohort studies: 3A, 16B, cross-sectional studies: 8B, 2C. Detailed information about all graded studies, divided by outcomes, is found in Appendices 3–8. Four papers had two separate outcomes.

The findings for each separate outcome are presented in Tables 2–8. Only the 34 studies graded A and B are included in these tables and used in the final grading of evidence.

**Table 2:** Outcome growth/BMI: comparison of protein intake levels and outcome

Papers seeing a positive association between protein intake and growth, and/or BMI						

Author, year (ref no.) Country Study design (study name if applicable)	Low intake mean±SD or median (25th–75th percentiles)	High intake mean±SD or median (25th–75th percentiles)	Age food	Effect	Age weight/BMI	Comment
Gunnarsdottir 2003, (3) nationwide longitudinal Iceland	Mean for group (E%): 15±4 at 9 months, 16±3 at 12 months		0–12 months (monthly) 24 h-records	Boys in the highest quartile of protein intake (E%) at the age of 9–12 months had significantly higher BMI (17.8±2.4 kg/m^2^) at 6 years than the lowest (15.6±1.0 kg/m^2^, *p*=0.039) and the second lowest (15.3±0.8 kg/m^2^, *p*=0.01) quartile.	6 year	Together, weight gain at 0–12 months and protein intake at 9–12 months explained 50% of the variance in BMI among 6-year-old boys.	
Günther, 2007 (34) Germany Cohort study DONALD	Median at 18–24 months E% 13.3 (11.8–14.7) g/kg/day 2,6 (2.3–3.0)	Median at 18–24 months E% 13.8 (12.9–15.2) g/kg/day 2.6 (2.4–3.0)	6, 12, 18–24 months 3-day weighed records	Consistently high protein intake 12, 18–24 months positively related to increased BMI SDS and %BF at 7 years; BMI SDS 0.37 (95%CI 0.12. 0.61) vs. 0.08 (-0.09, 0.26), *p*=0.04 %BF 18.37 (17.29, 1.51) vs. 16.91 (16.19,17.66), *p*=0.01 OR for BMI >75th percentile 2.39 (1.14, 4.99), *p*=0.02 OR for %BF >75th percentile 2.28 (1.06,4.88), *p*=0.03	7 year	
Günther, 2007 (35) Germany Cohort study DONALD	Median for group at 12 months: E% 13.3 (11.7–14.8) g/kg/day 2.7 (2.4–3.2)		6, 12, 18–24 months, 3–4 years, 5–6 years 3-day weighed records	Animal protein intake at 12 months positively assoc w %BF [mean/tertil [95% CI) T1; 16.2(15.23,17–25), T2; 17.21(16.24, 18.23), T3; 18.21(17.12, 19.15), *p*=0.008 Animal protein E% at 12 months positive association to BMI a 7 year (*p*=0.02) Dairy E% at 12 months, but not meat or cereal, positive association to BMI a 7 year (*p*=0.02) and %BF at 7 year (*p*=0.07).	7 year		
Hoppe, 2004 (36) Denmark Cohort (observational)	Median for boys at 12 months: E%:14 (9–18) g/kg/day: 2.7 (1.6–3.9) Median for girls at 12 months: E%:13 (9–17) g/kg/day: 2.8 (1.8–3.6)		9 months: 5 days’ weighed food records 10 year: 7 days’ food record	Effect estimates (linear regression) between protein intake 9 months and body composition (BMI and %BF) at 10 years: Protein E% at 9 months was a predictor for weight at 10 years: 0.44(0.12–0.76), *p*=0.007, and height 0.51 (0.13–0.90), *p*=0.009. Protein intake (g/day – but not g/kg/day) was a predictor for weight, 0.16(0.037–0.29), *p*=0.03	10 year		
Hoppe, 2004 (13) Denmark Cross-sectional	10th percentile 2.4 g/kg/day 50th percentile 2.9 g/kg/day	90th percentile 4.0 g/kg/day	2.5 year 7 days’ record	In multiple linear regressions with adjustment for sex and weight, height (cm) was positively associated with intakes of animal protein (g/day) [0.10±0.038 (*b*±SE); *p*=0.01] and milk (0.0047±0.002; *p* =0.007), but not with those of vegetable protein or meat.	2.5 year	Cross-sectional 63% was animal protein
Koletzko, 2009 (29) RCT Multi-center, European double-blind intervention trial	Infant formula 1.77 g protein/100 kcal follow-on formula 2.2 g protein/100 kcal	Infant formula 2.9 g protein/100 kcal follow-on formula 4.4 g protein/100 kcal	3, 6, 12 and 24 months 3-day record	A higher protein content of infant formula was associated with higher weight in the first 2 years of life but had no effect on length. At 24 months, adjusted z score for weight-for-length in the lower protein formula group was 0.20 (95% CI: 0.06, 0.34; *p*=0.005) lower than in the higher protein group and did not differ from that of the breastfed reference group.	24 months	For comparison, ‘exclusively breastfed’ also followed (<10% of feedings or <3 bottles of formula/week during first 3 months)
Kourlaba, 2008 (42) Greece Cross-sectional GENESIS	Mean intake normal weight (E%) 16.5±2.6	Mean intake over weight (E%) 16.6±2.5	1–5 year 3-day record	Protein intake was higher among ‘at risk of being overweight’ or ‘overweight’ compared with their normal-weight counterparts.	1–5 year	Cross-sectional
Manios, 2008 (44) Greece Cross sectional	g/day (Mean±SD) Normal weight 57.2±12.2 At risk OW 59.6±12.4 OW 59.9±12.8		1–5 year 3-day record	‘At risk of overweight’ and ‘overweight’ children consumed more total energy, protein, and fat compared with their normal-weight counterparts.	1–5 year	Cross-sectional Usual dietary intake at 1–5 years NOTE: no difference in E% Normal weight 17.1±1.6 At risk OW 17.1±1.5 OW 17.1±1.5	
Morgan, 2004 (37) UK Cohort	Mean (SD) g meat/day lower tertile 4–24 months: 16±4–35±9	Mean (SD) g meat/day upper tertile 4–24 months: 17±5–43±11	4, 8, 12, 16, 20 and 24 months (intake calculated as average intake 4–24 months) 7 days’ records	Meat intake from 4 to 12 months was positively and significantly related to weight gain (*p*< 0.05);	22 months	1930–40
Öhlund, 2010 (4) Sweden Prospective cohort study	Mean 17–18 months 4 E%: Boys/girls 13.6±1.6 g/kg/day: Boys: 2.9±0.6, girls 3.0±0.5: Mean 4 years E%: Boys: 13.4±1.8, girls 13.1±1.6 g/kg/day: Boys: 2.7±0.6, girls 2.6±0.5:		6–18 months + 4 years: Monthly 5 days’ records	protein intake at 17/18 months and at 4 years were positively associated with BMI at 4 years	4 year		
Sandström, 2008 (31) Sweden CT/Partly RCT	25% α-lactalbumin, with 15% glycomacropeptide GMP 25% α -lactalbumin, with 10% GMP	Standard formula:11% α-lactalbumin, 14% GMP	6 weeks–6 months	All formulas: 1,96 g prot/ 100 kcal. The standard formula group gained significantly more weight than did the breastfed infants.	6 months	No volumes given
Scaglioni, 2000 (38) Italy Prospective cohort	20 E%	22 E%	1 and 5 year Age-adjusted FFQ and 24H-recalls	Five-year old overweight children had a higher percentage intake of proteins at the age of 1 year than non-overweight children (22 vs. 20%, *p*=0.024)	5 year	Strongest risk factor for overweight at 5 years was parental overweight (*p*<0.0001).
Skinner 2004, (39) Prospective cohort	Mean 14 E% protein (longitudinal protein intake 2–8 years)		2 to 8 years. longitudinal intakes were based on 27 days of dietary data per child, collected in nine sets of 3 days’ data	mean protein and fat intakes recorded between 2 and 8 years were positive predictors of BMI at 8 years; mean carbohydrate intake over the same time period was negatively related to BMI at 8 years	8 year		
**Papers seeing no association between protein intake and growth and/or BMI**						
Reference details	Low intake	High intake	Age food	Effect	Age weight/BMI	Comment
Larnkjær, 2009 (30) RCT	Infant formula 11.4 E% protein at 12 months	Whole milk 14.2 E% protein at 12 months	9 and 12 months	No effect of the milk intervention on change in weight or length.	9 and 12 months	
Maillard 2000 (43) France Cross-sectional	Mean (range) of E% at ages 5–11 years (age and height adjusted) Boys: 14.0 (5.5–23.8) Girls: 14.3 (7.4–23.5)		1-day record	No association related to E% protein	5–11 year	Cross-sectional	
Räihä 2002 (5) CT/RCT	Two experimental formulas with a whey/casein ratio of 70/30 and a protein content of 1.8 g/ 100 kcal.	A conventional whey adapted starter formula with a whey/casein ratio of 60/40 and a protein content of 2.2 g/100 kcal	3-day food record	No differences were found between the four feeding groups for weight- and length-gains or for body mass indices (BMI). No differences in energy intakes between the formula fed groups could be found, whereas protein intakes were less in infants fed the 1.8 g/100 kcal formulas.	30–120 days of age	
Van Vught, 2010 (40) Denmark Prospective cohort	E% Boys: 69.5±17.8 Girls: 63.4±15.2 1–4th quintile of BMI (leaner children)	E% Boys: 71.3±15.7 Girls: 59.7±13.7 5th quintile of BMI (heavier children)	7-day records at 6 years	No association between protein intake and linear growth. However, amino acids could be important. High arginine (ARG) intake, but not lysine (LYS), was associated with linear growth (β = 1.09 (se 0.54), *p*= 0.05) among girls. Also in girls, change in fat mass index (FMI) showed a stronger inverse association if combined with high LYS, compared with low LYS intake.	6 and 9 years	Possibly association with amino acids rather that protein as such

All steps in the process of selecting and grading papers, that is, abstract screening, paper screening, and quality assessment, were performed as described in the guide for conducting SLRs (26). This meant that two experts evaluated each abstract/paper. The experts first made an individual appraisal, which was then discussed and a joint conclusion was decided upon.

The grade of evidence was classified as convincing (grade 1), probable (grade 2), limited-suggestive (grade 3), and limited-inconclusive (grade 4) depending on the number and quality of supporting, non-supporting, and contradicting studies.

## Results

The majority of studies (23/34) on protein intake in infancy and childhood had different aspects of growth and body weight as outcomes (Table 2). Only a few studies focused on puberty, blood pressure, and neurodevelopment making the evidence very weak for any conclusions. When papers originated from the same research group, it was not always possible to tell whether or not the participating children were the same in several studies. This is problematic as evidence grading requires evidence from at least two *independent* cohort studies, or at least five case-control studies. We have taken this into account in our grading.

### BMI, growth, body composition, and sIGF-I

Tables 2–3 show summaries of studies with outcome BMI, growth, body composition, and sIGF-I (details about the studies are provided in Appendix 3). In total, 23 papers were chosen in the systematic review process to be evaluated for the evidence of an association between protein intake and these outcomes. Sixteen of those include outcomes on BMI/growth and these are briefly described in Table 2, in addition to the more detailed descriptions given in Table 3 and Appendix 3 for all the 23 studies. Of these studies, 6 were clinical trials (1A, 5B), 12 were prospective cohort studies (2A, 10B), and 5 were cross-sectional studies (all B). One additional cross-sectional study graded C was excluded.

**Table 3:** Protein intake and outcome BMI, growth, body composition, IGFS-I (6 clinical trials, 12 cohort, 5 cross-sectional)

Author, year (ref no.) Country Study design (study name if applicable)	No. of participants	Exposure (incl age)	Outcome (incl age)	Effect/association	Study quality comments
Budek, 2007, (14) Denmark, Cross-sectional	84 out of 96 boys (84%)	Intake of total, dairy and meat protein at 8 years	Concentrations of sIGF-I and markers for bone-turnover; serum osteocalcin (s-OC), bone-specific alkaline phosphatase (s-BAP) and C-terminal telopeptides of type l collagen (s-CTX) at 8 years	Dairy protein was negatively associated with sOC (*p*=0.05) but not significantly associated with sBAP and sCTX. Dairy protein decreased (*p*=0.05) sOC at a high meat protein intake (>0.8 g/kg), whereas meat protein increased (*p*=0.03) sOC at a low dairy protein intake (<0.4 g/kg). Total and meat protein intake was positively associated with sBAP (*p*≤0.04) but not significantly associated with sOC and sCTX. Free sIGF-I was positively associated with total (*p*<0.01) and dairy (*p*=0.06) protein but not with meat protein.	B Results not adjusted for mis/underreporting, no power calculation. Definition of meat protein includes meat, poultry and fish (but not pork, egg)
Dorosty, 2000, (32) UK Prospective cohort (ALSPAC)	772 out of 889 (87%)	Protein intake at 18 months (g/day and E%)	Timing of adiposity rebound (AR) (hypothesis: high protein intake promotes early AR)	No evidence of associations between protein intake, or any other dietary variable, and timing of the AR. Children with AR very early (≤ 43 months) or early (from 49 but before 61 months) had parents with sig higher BMI and were sig more likely to have at least 1 obese parent.	B Measurement errors in dietary recording not considered, very little data given about recordings, power calculation not done,
Gunnarsdottir 2003, (3) Iceland Nationwide longitudinal cohort	90 children (41 boys)	Size at birth, growth and food intake in infancy	BMI at 6 years (Weight and height were measured at maternity wards and healthcare centers in Iceland throughout infancy and at 6 years)	Weight gain from birth to 12 months as a ratio of birth weight was positively related to BMI at the age of 6 years in both genders (β = 2.9±1.0, *p*=0.008, and β = 2.0±0.9, *p*=0.032 for boys and girls, respectively). Boys in the highest quartile of protein intake (E%) at the age of 9–12 months had significantly higher BMI (17.8±2.4 kg/m^2^) at 6 years than the lowest (15.6±1.0 kg/m^2^, *p*=0.039) and the second lowest (15.3±0.8 kg/m^2^, *p*=0.01) quartile. Energy intake was not different between groups. Together, weight gain at 0–12 months and protein intake at 9–12 months explained 50% of the variance in BMI among 6-year-old boys. Rapid growth during the first year of life is associated with increased BMI at the age of 6 years in both genders. In boys, high intake of protein in infancy could also contribute to childhood obesity.	B Measurement errors in dietary recording not considered, power calculation not done
Günther, 2006 (33) Germany Cohort study DONALD	313 children with complete data (161 boys, 152 girls) up to 7 years	Habitual energy adjusted protein intake (E% and g/kg RBW/day, average between 2–3 dietary records between 12 and 24 months. RBW = reference body weight (adjusted for age- and sex-specific)	Timing of adiposity rebound (AR) (hypothesis: high protein intake promotes early AR and higher BMI at AR)	After adjusting for potential confounders, girls in the highest tertile (T3) of habitual energy-adjusted protein intake had a significantly higher BMI-SDS at AR than those in T1 (T1:-0.61 (95% CI: -0.90; -0.31), T2:-0.49 (-0.79; -0.20), T3:-0.08 (-0.36; 0.20), *p* for difference = 0.01). A comparable association existed with habitual protein intake expressed as g/kg RBW/day (T1:-0.64 (-0.93; -0.36), T2:-0.22 (-0.52; 0.09), T3: -0.25 (-0.54; 0.04), *p*=0.04). In boys, there were no differences in BMI-SDS at AR between tertiles of habitual protein intake (% of energy or g/kg RBW/day) (P40.05). Boys in the lowest tertile of habitual energy-adjusted protein intake tended to experience a later AR (T1: 6.0 (5.6; 6.4), T2: 5.5 (5.1; 5.9), T3: 5.4 (5.0; 5.9) years, *p*=0.07). But neither in girls nor in boys was age at AR significantly different between tertiles of habitual protein intake (% of energy or g/kg RBW/day) (*p*>0.05). A higher habitual protein intake between the age of 12 and 24 months was associated with a higher BMI-SDS at AR in girls, but not in boys. There was no consistent relation between habitual protein intake in early childhood and timing of AR.	B Measurement errors in dietary recording not considered, power calculation not done
Günther, 2007 (34) Germany Prospective cohort, DONALD Study	203 (104 M, 99 F)	Protein intake at 6, 12 and 18–24 months	BMI and %BF (per cent body fat) at 7 years of age	↑protein →↑BMI Consistently high protein intake 12, 18–24 months positively related to increased BMI SDS and %BF at 7 years; BMI SDS 0.37 (95% CI 0.12. 0.61) vs. 0.08 (-0.09, 0.26), *p*=0.04; %BF 18.37 (17.29, 1.51) vs. 16.91 (16.19,17.66), *p*=0.01. OR for BMI >75th percentile 2.39 (1.14, 4.99), *p*=0.02; OR for %BF >75th percentile 2.28 (1.06, 4.88), *p*=0.03. No effect of protein intake at 6 months.	A Reported energy intake a bit low in one group (<−20% of standard for age for the low-low group, Table 2)
Günther, 2007 (35) Germany Prospective cohort, DONALD Study	203 (102 M, 101 F)	Protein intake at 6, 12, 18–24 months, 3–4 years, 5–6 years	BMI and %BF (per cent body fat) at 7 years of age	12 months and 5–6 years identified as critical periods at which higher total and animal, but not vegetable, protein intakes were positively related to body fatness at 7 years. Animal protein intake at 12 months positively associated with %BF [mean/tertil [95% CI) T1; 16.2(15.23,17–25), T2; 17.21(16.24, 18.23), T3; 18.21(17.12, 19.15), *p*=0.008 Animal protein E% at 12 months positive association to BMI a 7 years (*p*=0.02) Animal protein E% at 12 months and 5–6 years positive association to %BF at 7 years (*p*=0.01). Dairy E% at 12 months, but not meat or cereal, positive association to BMI a 7 years (*p*=0.02) and %BF at 7 years (*p*=0.07).	A 2.6% records excluded (potentionally implausible FIL/PAL) Median energy intake just above <20% so credible but a bit low.
Hoppe, 2009, (27) Denmark CT	57 boys	At 8 years a 7-day intervention with 540 ml milk-based drinks, either: 1) whey with low mineral content (Ca and P) (Whey-low), 2) whey with high mineral content (Whey-high), 3) casein with low mineral content (Case-low), 4) casein with high mineral content (Case-high)	Serum IGF-1, IGFBP, fasting insulin, C-peptide, index of insulin resistance, glucose	No interactions between milk mineral groups (high, low) and milk protein groups (whey, casein). The milk protein intervention groups were combined. Average daily protein intake was increased by 17% by the whey drink, from 58 g/day (2.23 g/kg per day, 12.98 PE%) to 68 g/day (2.56 g/kg per day, 15.42 PE%) (*p*<0.001), and by 51% by the casein drink, from 68 g per day (2.30 g/kg per day, 14.30 PE%) to 103 g per day (3.44 g/kg per day, 23.40 PE%) (*p*<0.001). In the whey group, fasting insulin increased by 21% (*p*=0.006), with no change in IGF-1 (*p*=0.27). In the casein group, serum IGF-1 increased by 15% (*p*<0.0001), whereas there was no change in fasting insulin (*p*=0.36).No independent effects of a high milk mineral intake on IGF-1 and insulin. Increase in serum urea nitrogen (SUN), and the molar ratio of IGF-1/IGFBP-3 was significantly higher in the combined casein-group than in the combined whey group. Conversely, whey increased fasting insulin more than did casein.	B 36% drop-out. No details given. Remaining diet unclear. Energy intake at baseline reported and credible level. Nothing said about 7-day Measurement errors not considered Can′t find that they say very much about compliance.
Hoppe, 2004 (28) Denmark Intervention study (7-day)	24 boys	At 8 years a 7-day intervention with 53 g protein daily, 12 boys as 1.5 l skimmed milk, and 12 boys as 250 g low fat meat. In addition, they were asked to eat their normal diet ad libitum.	IGF-I concentrations and the molar ratio of IGF-I/IGFBP-3 in healthy, prepubertal children	After 7 days, the average protein intake increased in milk group by 61%; meat group +54%. The milk group increased s-IGF-I by 19% (*p*=0.001) an s-IGF-I/s-IGFBP-3 by 13% (*p*<0.0001). No increase in the meat group. Conclusion: Compounds in milk and not a high protein intake as such seem to stimulate IGF-I. This might explains the positive effect of milk intake on growth seen in some studies.	B No power calculation Compliance is not reported. It is stated that protein intake is increased but nothing about how much of the additional 15 dl of milk/250 g of meat was actually taken, and very little about how their habitual diet changed.
Hoppe, 2004 (36) Denmark Prospective, observational cohort	142 with data from 9 months invited to 10 years follow-up, 105 (74% agreed to take part), 51 M and 53 F (+ 1?)	Protein intake at 9 months, and 10 years Protein intake (as measured by SUN (serum urea nitrogen) and IGF-I at 10 years	Weight and height at 10 years	SUN at 9 months was a predictor for weight at 10 years: 0.96 (0.28–1.6), *p*=0.006Protein E% at 9 months was a predictor for weight at 10 years: 0.44 (0.12–0.76), *p*=0.007, and height 0.51(0.13–0.90), *p*=0.009.Protein intake (g/day – but not g/kg/day) was a predictor for weight, 0.16 (0.37–0.29), *p*=0.03 [Wrong in table. Should be 0.16 (0.037–0.29)] and height 0.19(0.042–0.34), *p*=0.003.	B Measurement errors in dietary recording not considered, power calculation not done
Hoppe, 2004 (13) Denmark cross-sectional	90 children (54 boys, 46 girls)	Protein intake (g/kg/day) at 2.5 years	Associations between protein intake, serum insulin-like growth factor I (sIGF-I) concentrations, and height in in 2.5-year-old healthy children.	The 10th, 50th, and 90th percentiles of protein intake were 2.4, 2.9, and 4.0 g/kg/day, respectively; 63% was animal protein. In multiple linear regressions with adjustment for sex and weight, height (cm) was positively associated with intakes of animal protein (g/day) [0.10±0.038 (*b*±SE); *p*=0.01] and milk (0.0047±0.002; *p*=0.007), but not with those of vegetable protein or meat. The sIGF-I concentration was significantly associated with intakes of animal protein (1.4±0.53; *p*=0.01) and milk (0.049±0.024; *p*= 0.045), but not with those of vegetable protein or meat. sIGF-I concentrations were positively associated with height (0.019±0.008; *p =* 0.02). Milk intake was positively associated with sIGF-I concentrations and height. An increase in milk intake from 200 to 600 mL/day corresponded to a 30% increase in circulating IGF-I. This suggests that milk compounds have a stimulating effect on sIGF-I concentrations and, thereby, on growth.	B Measurement errors in dietary recording not considered, power calculation not done
Koletzko, 2009 (29) European Multicenter study RCT	Children in five countries (Belgium, Germany, Italy, Poland and Spain), *n*=934 followed until 24 months; 636 in the lower (*n*=313) and higher (*n*=323) protein formula groups and 298 in the breastfed group.	Infant formula and follow-on formulas with a lower (1.77 and 2.2 g protein/100 kcal, resp) or higher (2.9 and 4.4 g protein/100 kcal, resp) content of cow milk protein. For comparison, ‘exclusively breastfed’ also followed (<10% of feedings or <3 bottles of formula/week during first 3 months)	Primary outcome: Length, weight at 24 months, expressed as length and weight-for-length z scores based on 2006 WHO growth standards. Secondary outcome: weight, length, weight-for-length and BMI at inclusion, at 3, 6, 12 and 24 months	↑protein →↑weight A higher protein content of infant formula was associated with higher weight in the first 2 year of life but had no effect on length. At 24 months, adj. z score for weight-for-length in the lower protein formula group was 0.20 (95% CI: 0.06, 0.34; *p*=0.005) lower than in the higher protein group and did not differ from that of the breastfed group. In general, differences were greatest at 12 months for weight, weight-for-length and BMI. Compared with breastfed, those fed high-protein formula had sig higher z scores for weight, length, weight-for-length and BMI at 24 months. Intervention effect did not differ between countries.	A parents lost to follow-up had lower education, mothers more likely to be smokers, BUT no difference between study arms. No differences for those excluded due to non-compliance Biomarkers not used
Kourlaba, 2008 (42), GENESIS Cross-sectional	Uncertain about final number analysed; between 2033 and 2346	Energy and macronutrient intake, including protein, children 1–5 years	Interaction effect between angiotensin-converting enzyme 1 (ACE) 1/D polymorphism and diet on obesity-related phenotypes. DNA samples from 2102 children (1–5 years) were genotyped for the *ACE* I/D polymorphism; 3 genotypes (ll, lD, DD)	↑protein →↑BMI Significant interactions found between the *ACE* I/D polymorphism and protein intake on BMI and being overweight (*p*<0·05 for interaction). Stratified analyses revealed that total energy is correlated with WC and protein intake is associated with BMI and being overweight only among carriers of the D-allele (i.e. DD or ID genotypes). Protein intake was found to be positively associated with the likelihood of being overweight and with BMI (marginally) among the DD homozygotes. Protein intake was higher among ‘at risk of being overweight’ or ‘overweight’ compared with their normal-weight counterparts.	B No power calculation (They′ve used Bonferroni correction, but it is not clear how)
Larnkjær, 2009 (30) Denmark Randomized trial	Healthy infants (*n*=83) randomized to either whole milk (WM) or infant formula (IF)	Infants randomized to either WM or IF (and either a daily fish oil supplement or no supplement. (2×2 design)	Weight and length at 9 and 12 months and increase in weight and length. IGF-l (insulin-like growth factor l)	WM or IF no effect on change in weight and length. Intake of WM sign increased the protein energy percentage (PE%; *p*<0.001) and SUN (*p*=0.01). PE% was 14.2 in WM and 11.4 in IF at 12 months. But no effect of the milk intervention on change in weight or length. Intake of fish oil had no effect on the outcomes.	B Dropout infants did not differ in birth or breast-feeding characteristics from those who finished the study, but they were 1.6 cm shorter at 9 months (95% CI 3.01, 0.21, *p*=0.039). This could be problematic as it is a study about growth.
Maillard, 2000 (43) France Cross-sectional	501; 280 boys, 221 girls (aged 5±11 years)	Dietary intake at 5–11 years Energy, protein and other nutrients RQ: associations between several adiposity indices and the nutrient intake The associations were looked for according to current dietary recommendations, and according to reported energy intake to basal metabolic rate ratios (EI=BMR) and gender.	Height and weight, four skinfolds (biceps, triceps, subscapular, suprailiac), waist and hip girths, were measured. Sum of skinfolds (SSF), body mass index (BMI), and relative weight (RW) were calculated. Energy intake (EI), percentage of energy intake ascribed to carbohydrates (%EIC), complex carbohydrates (%EICC), fats (%EIF), saturated fats (%EISF) and proteins (%EIP)	In multiple linear regressions analyses performed with hierarchical mixed models, adiposity indices were significantly and inversely associated in girls with %EIC (all *p*-values < 0.02), and positively with %EIF (all *p*-values <0.05, waist girth and BMI excepted). Similar but non-significant trends were observed in boys. The relationships were not linear, and thresholds close to current dietary recommendations were highlighted. When%EIF was low, a lower percentage of energy intake ascribed to%EISF was associated with thinness. These associations remained after the exclusion of children who had an EI=BMR _1.50. For both fat and carbohydrate, a substantial percentage of toddlers and preschoolers had usual intakes outside the acceptable macronutrient distribution range, whereas protein was less than this range. ‘At risk of overweight’ and ‘overweight’ children consumed more total energy, protein, and fat compared with their normal-weight counterparts, whereas no differences were found for micronutrient intakes. The estimated prevalence of inadequacy was found to be between 10 and 25% for niacin, vitamin E, and folate. Usual intakes exceeding the Tolerable Upper Intake Levels were recorded for zinc and copper.	B Confounders not taken adequately into consideration
Manios, 2008 (44) Greece Cross-sectional (GENESIS cohort)	2374, age 1 to 5 years	Describe nutrient intake: (a) usual energy and macronutrient intake in the total population as well as by children's weight status, and (b) inadequate or excessive nutrient intakes compared with children's requirements.	Anthropometrical indexes (i.e. body weight, recumbent length, and stature) obtained and BMI was calculated The Nutstat module of EpiInfo was used to determine children's age- and sex-specific percentiles for weight, length, and body mass index. The weight-for-length percentiles were used to classify children up to 24 months old as ‘overweight’ (≥95th percentile), whereas children older than 24 months were classified as ‘at risk of overweight’ (≥85th and <95th percentile) and ‘overweight’ (≥95th percentile) using the body mass index-for-age percentiles.	For both fat and carbohydrate, a substantial percentage of toddlers and preschoolers had usual intakes outside the acceptable macronutrient distribution range, whereas protein was less than this range. ‘At risk of overweight’ and ‘overweight’ children consumed more total energy, protein, and fat compared with their normal-weight counterparts, whereas no differences were found for micronutrient intakes. The estimated prevalence of inadequacy was found to be between 10 and 25% for niacin, vitamin E, and folate. Usual intakes exceeding the Tolerable Upper Intake Levels were recorded for zinc and copper.	B Participation rate not clear, study power not reportedNOTE: no difference in protein E% between the groups (normal weight 17.1±1.6, at risk OW 17.1±1.5, OW 17.1±1.5)
Morgan, 2004 (37) UK Prospective cohort	144	1. Total red and white meat intake (g) from 4 to 12 months as a continuous variable, i.e. total meat intake over 21 days between 4 and 12 months.2. Total red and white meat intake (g) from 4 to 16 months as a continuous variable, i.e. total meat intake over 28 days between 4 and 16 months.3. Total red and white meat intake (g) from 4 to 24 months as a continuous variable, i.e. total meat intake over 42 days between 4 and 24 months.	Body weight, length, and head circumference at the ages of 4, 8, 12, 16, 20 and 24 months	Meat intake from 4 to 12 months was positively and significantly related to weight gain (*p*<0.05); further analysis suggested this association might be mediated via protein intake but was independent of energy, zinc or iron intake. There was no interaction between meat intake and breastfeeding on growth. These findings remained after adjustment for potential confounding factors.	B Loss to follow-up not reportedNo power calculations
Öhlund, 2010 (4) Sweden Prospective cohort study	127 healthy children (63 girls and64 boys) at 4 years of age followed prospectively from 6 to 18 months of age	Current and previous dietary intake	Weight, height BMI, Mid-upper arm circumference, subcutaneous fat at 4 years of age	Fourteen percent of the girls and 13% of the boys were overweight (age-adjusted BMIX25) and 2% of the girls and 3% of the boys were obese (age-adjusted BMIX30). Thirty-four percent and 9% of the fathers and 19 and 7% of the mothers were overweight and obese, respectively. BMI at 6–18 months was a strong predictor of BMI at 4 years. Intake of protein in particular, and also of total energy and carbohydrates at 17/18 months and at 4 years, was positively associated with BMI at 4 years. Although BMI at 6–18 months was the strongest predictor of BMI at 4 years, in the final multivariate models of the child's BMI, protein intake at 17–18 months and at 4 years, energy intake at 4 years and the father's—but not the mother's—BMI were also independent contributing factors	B Loss of follow-up more than 20% No power calculation
Räihä, 2002 (5) Finland CT	113 term infants, breast-fed and formula-fed.	Parents were instructed to exclusively breastfeed or feed the assigned formula up to 120 days of age (3 isocaloric formulas differing by their protein source and content were studied and compared with breast milk) Calculated energy and protein intakes	increment in anthropometrics parameters from 30 up to 120 days of age (unit/month). Body weight and length were obtained at birth, at 30, 60, 90, and 120 days. Blood was collected for biochemical measurements at 30, 60, and 120 days.	No differences were found between the four feeding groups for weight- and length-gains or for body mass indices (BMI). No differences in energy intakes between the formula fed groups could be found, whereas protein intakes were less in infants fed the 1.8 g/100 kcal formulas. Plasma urea levels of the infants fed the 1.8 g/100 kcal formulas were closer to those found in the breast-fed infants.	B Randomization method not stated adequately Differences between drop-outs and participants nor reported
Sandström, 2008 (31) Sweden CT/Partly RCT	80 (HealthyGA: 36–42 weeksBWT: 2500–5000 g)	Standard vs. two formulas varying in G Lycomacropeptide (GMP) and α-lactalbumin i.e. 3 formulas w. bovine whey fractions rich in α-lactalbumin w. varying GMP vs. breast feeding (as control) All formulas: 1,96 g prot/ 100 kcal.	-Growth -General health -Plasma leptin, insulin, urea nitrogen, amino acids	Formula intake was similar in different groups. Weight gain in the alpha-lactalbumin-enriched formula groups was similar to that of the breastfed infants. The standard formula group gained significantly more weight than did the breastfed infants. All formula-fed infants had significantly higher plasma concentrations of most essential amino acids at 4 and 6 months than did the breastfed infants, and serum urea nitrogen was also higher in the formula-fed infants. Insulin and leptin concentrations did not differ between groups.	B No power calculation reported, compliance unclear, energy intake unclear, results not analysed blind, unclear about between measurements errors
Scaglioni, 2000 (38) Italy Prospective cohort	147	Nutrients/ early macronutr. Intake, Parental factors	Anthropometry at 1, 5 years	The prevalence of overweight at the age of 5 years was strongly associated with parental overweight (*p*<0.0001). Five-year old overweight children had a higher percentage intake of proteins at the age of 1 year than non overweight children (22 vs. 20%, *p*=0.024). Multiple logistic analysis confirmed that protein intake at 1 year-of-age was associated with overweight at 5 years (*p*=0.05). In children born from overweight mothers, prevalence of overweight at the age of 5 years tended to be higher in bottle-fed than in breast-fed ones (62.5 vs. 23.3%, *p*=0.08). Conclusion: Parental overweight is a major risk factor for childhood overweight in the first years of life, but an early high protein intake may also influence the development of adiposity.	B Measurement errors in dietary reporting not considered Energy intake little bit high No power calculation
Skinner 2004, (39) Prospective cohort	70	Energy and macronutrient intakes at each study point	BMI, age of adiposity rebound was determined	Children's BMI at 8 years was negatively predicted by age of adiposity rebound and positively predicted by their BMI at 2 years. Mean protein and fat intakes recorded between 2 and 8 years were positive predictors of BMI at 8 years; mean carbohydrate intake over the same time period was negatively related to BMI at 8 years. R2 values indicated that these three-variable models predicted 41–43% of the variability in BMI among children. BMI of 23% of the children exceeded the 85th CDC percentile.	B Baseline not clearly indentified, easurement errors in dietary recording not considered, power calculation not done
Van Vught, 2010 (40) Denmark Prospective cohort	223	Protein intake, especially the amino acids: Lysine (LYS) Arginine (ARG)	Growth & body composition Fat mass index (FMI)	No association between protein intake and linear growth. However, amino acids could be important. High ARG intake, but not LYS, was associated with linear growth (β = 1.09 (se 0.54), *p*=0.05) among girls. Furthermore, in girls, change in FMI had a stronger inverse association with high ARG intake, if it was combined with high LYS intake, instead of low LYS intake (*p*=0.03). No associations were found in boys. In prepubertal girls, linear growth may be influenced by habitual ARG intake and body fat gain may be relatively prevented over time by the intake of the amino acids ARG and LYS.	B Energy adjustment not done No power calculation
Van Vught, 2009 (41) Denmark Prospective cohort	384 of originally 771	Diet Protein, amino acids: ARG, LYS	Skinfold thickness was measured at ages 8–10 years and 14–16 year. BMI and Body fat% was estimated from skinfold measurements	Among lean girls inverse associations were found between protein as well as arginine and lysine intake and change in fat mass index (β = −1.12 + /−0.56, *p*=0.03, β = −1.10 + /−0.53, *p*=0.04, β = −1.13 + /−0.51, *p*=0.03 respectively). Furthermore among girls with a body mass index in the 5th quintile, protein intake was associated with DeltaFFMI (*p*=0.04), and more specific when LYS intake was high, ARG intake was associated with DeltaFFMI (*p*=0.04). No associations were found in boys.	B Measurement errors in dietary reporting not consideredOnly 49.8% of original sample took part in baseline (57% of those still living in area took part in follow-up)

#### Clinical trials

Hoppe et al. (27), graded B, examined in a double-blinded randomized trial (*n*=59) the effects of the two major milk fractions, whey and casein, and milk minerals, calcium (Ca) and phosphorus (P), on sIGF-I and glucose–insulin metabolism (see results in “Glucose–insulin metabolism”). Eight-year-old boys were randomized to receive 540 ml of milk-based drinks for 7 days, either: 1) whey with low mineral content (Ca and P); 2) whey with high mineral content; 3) casein with low mineral content; and 4) casein with high mineral content. The amount of other milk components aimed to be identical to the content in 1.5 L of skimmed milk.

Dietary intake was assessed using two repeated 3-day weighed food records (2 weekdays and 1 weekend day); the first kept before the intervention (days −3 to 0) and the second at the end of the intervention (days 5 to 7). Measurement errors in the dietary recordings were not considered, but the importance of maintaining usual dietary intake was emphasized to the families. Unintentionally, there were significant differences between the intervention groups with regard to several of the anthropometrical measurements, energy and milk intake. No interactions between milk mineral groups (high, low) and milk protein groups (whey, casein) were found, so the milk protein groups were combined.

Average daily protein intake was increased by 17% by the whey drink, from 58 g/day (2.23 g/kg/day, 13.0 PE%) to 68 g/day (2.56 g/kg/day, 15.4 PE%), and by 51% by the casein drink, from 68 g/day (2.30 g/kg/day, 14.3 PE%) to 103 g/day (3.44 g/kg/day, 23.4 PE%). In the whey group, there was no change in sIGF-I. No independent effects of a high milk mineral intake on sIGF-I were found. Increase in serum urea nitrogen (SUN), a marker for protein intake, and the molar ratio of sIGF-I/sIGFBP-3 was significantly higher in the casein group than in the whey group.

A limitation of the study, also mentioned by the authors, is that the subjects were allowed to eat their habitual diet, so there might be other factors in the diet contributing to the findings. However, the authors point out that this has been controlled for in the analyses. The results were not changed markedly after controlling for energy intake, protein intake, SUN, or milk intake. The authors conclude that casein stimulates circulating sIGF-I and that both milk protein fractions seem to be important, but different, in the growth-stimulating effect of milk.

Hoppe et al. (28), graded B, conducted an intervention study (*n*=24) to examine whether an increase in animal protein intake could increase concentrations of sIGF-I in serum(s) and the molar ratio of sIGF-I/sIGFBP-3 in pre-pubertal children. Eight-year-old boys with a habitual milk intake of at least 500 ml/day were asked to take an extra 53 g protein daily for a week – extra daily intake was 1.5 l of skimmed milk by 12 boys and 250 g of low fat meat by 12 other boys. In addition, they were asked to eat their normal diet ad libitum. Dietary intake was assessed using two repeated 3-day weighed food records (2 weekdays and 1 weekend day); the first kept before the intervention (days −3 to 0) and the second at the end of the intervention (days 5 to 7). Measurement errors in the dietary recordings were not considered, but the importance of maintaining usual dietary intake was emphasized to the families.

After 7 days, the average protein intake increased to 4.0 g/kg/day (+61%) in the milk group and to 3.7 g/kg/day (+54%) in the meat group. High intake of milk increased concentrations of sIGF-I (+19%) and sIGF-I/sIGFBP-3 (+13%), while no increases were seen in the meat group. The authors conclude that compounds in milk, and not a high protein intake as such, seem to stimulate sIGF-I, and that this might explain the effect of milk intake on growth seen in some studies.

Note: The total reported energy intake by the milk group increased by 13% and the group gained on average 550 g of weight during the intervention week compared with a 3% increase in energy intake and no change in weight in the meat group. SUN was used as a biomarker of protein intake, but SUN increased similarly in the two groups although reported protein intake per kg and day increased 20% more in the milk group (+1.7 g/kg/day vs. +1.4 g/kg/day in the meat group). There was no difference in intake at baseline (2.32 g/kg/day vs. 2.27 g/kg/day). No power calculation was reported.

Koletzko et al. (29), graded A, conducted a double-blind, randomized controlled trial in a European multicenter study in five countries to test the hypothesis that a higher early protein intake leads to more rapid growth in the first 2 years of life. Eight-week-old healthy formula-fed infants (*n*=1138) were randomly assigned to a low- or high-protein diet (cow-milk-based infant formula with 1.77 vs. 2.9 g protein/100 kcal and follow-on formula with 2.2 vs. 4.4 g protein/100 kcal). The protein content represented approximately the lowest and highest acceptable levels in the range given in the European Union (EU) directives from 1991. An observational group of infants exclusively breastfed for the first 3 months of life was also included in the study (*n*=619) (Note: Exclusive breastfeeding was defined as <10% of feedings or less than three bottles of formula/week).

Dietary intake was assessed by 3-day weighed records during three consecutive days (2 weekdays and 1 weekend day) at 3, 6, 12, and 24 months. Energy intake was not calculated for food records containing any breastfeeding as breast milk intake was only measured in a subgroup. Food records with energy intake greater than three SDs of the mean by months and those deemed incomplete or with reported concurrent illness were excluded. (Note: No details given about the number of excluded records.)

Infant formula with higher protein content was associated with higher weight in the first 2 years of life but had no effect on length. At 24 months, adjusted z-score for weight-for-length in the lower protein formula group was 0.20 (95% CI: 0.06, 0.34; *P*=0.005) lower than in the higher protein group and did not differ from that of the breastfed reference group. The effect of intervention was not different between countries. Compared with breastfed children, those fed high-protein formula had significantly higher z-scores for weight, length, weight-for-length, and BMI at 24 months. Analyses were also performed at 3, 6, and 12 months, and in general, the differences were greatest at 12 months for weight, weight-for-length, and BMI. The authors conclude that limiting dietary protein intake during infancy might be a way to decrease the risk of overweight and obesity in later life.

Larnkjær et al. (30), graded B, performed a randomized controlled trial to study the effects of whole milk and infant formula on growth and IGF-l from 9 to 12 months of age in Denmark. Healthy infants (*n*=83) were randomized to receive either whole milk (WM) or infant formula (IF) and either a daily fish oil supplement or no supplement. Dietary intake was measured over seven consecutive days at 9 and 12 months using a pre-coded dietary record developed for children with portion sizes estimated from a portion size photo series. The infants in both groups consumed about 300 ml WM or IF per day. Measurement errors in the dietary records were not considered.

No effect of milk type on growth was found in this 3-month intervention study. [Note: Dropouts (17% of WM and 6% of IF) were 1.6 cm shorter at 9 months, which could be problematic as this was a study on growth in infancy. Also, the breastfeeding prevalence between 9 and 12 months was lower in the IF group, although including breastfeeding in the analysis made no difference.] Intake of WM significantly increased the PE%; PE% was 14.2 in WM and 11.4 in IF at 12 months, whereas no differences were found in weight and length between groups. The authors suggest that this could be due to the relatively short intervention period, and that there is a need for studies on the long-term effects of protein and milk protein.

Intake of WM increased sIGF-I in boys (*P*=0.034) but not in girls. Intake of fish oil had no effect on the outcomes. Including all infants, PE% was positively associated with sIGF-I at both 9 and 12 months after adjusting for sex and breastfeeding. Positive correlation was also found between sIGF-I and intake of WM and WM products adjusted for sex and duration of full breastfeeding. Boys had lower levels of sIGF-I and sIGFBP-3 at 9 and 12 months than girls. The authors conclude that the results suggest that high protein intake is positively associated with sIGF-I concentration in well-nourished infants.

Räihä et al. (5), graded B, studied breastfed and formula-fed healthy term infants. Infants who stopped breastfeeding before 28 days of age were randomly assigned to receiving one of the three study formulas up to 120 days of age. The three isocaloric formulas differed by their protein source: a conventional whey adapted starter formula with a whey/casein ratio of 60/40 and a protein content of 2.2 g/100 kcal was compared with two experimental formulas with a whey/casein ratio of 70/30 and a protein content of 1.8 g/100 kcal.

No differences were found between the four feeding groups for weight- and length-gains or for body mass indices (BMI). No differences in energy intakes between the formula-fed groups could be found, whereas protein intakes were lower in infants fed the 1.8 g/100 kcal formulas. Plasma urea levels of the infants fed the 1.8 g/100 kcal formulas were closer to those found in the breastfed infants. The authors conclude that a whey predominant formula with a protein/energy ratio of 1.8 g/100 kcal provides adequate intakes of protein from birth to 4 months of age.

Sandström et al. (31), graded B, did a (partly) randomized control study on the effects on growth of exchanging part of the protein in milk-based formulas with α-lactalbumin. All formulas contained 1.96 g protein/100 kcal, but with differences in the protein composition. Breastfed infants were controls. Weight gains in the α-lactalbumin-enriched formula groups were similar to that of the breastfed infants. The standard whey-predominant formula group gained significantly more weight than did the breastfed infants. All formula-fed infants had significantly higher plasma concentrations of most essential amino acids at 4 and 6 months than did the breastfed infants, and SUN was also higher in the formula-fed infants, which might indicate that the protein content of α-lactalbumin-enriched formula can be further reduced.

#### Prospective cohort studies

Dorosty et al. (32), graded B, studied the adiposity rebound (AR) among 5-year-olds in relation to protein intake at 18 months. Dietary intake was assessed through 3-day household measured records at 18 months (records were also collected at 8 months but were not used in the present study). Measurement errors in the dietary recordings were not considered. No evidence for an association between protein intake, or any other dietary variable, and timing of the AR were found. Children with very early (≤43 months) or early (from 49 but before 61 months) AR had parents with significantly higher BMI and were more likely to have at least one obese parent.

Gunnarsdottir et al. (3), graded B, studied BMI at 6 years in relation to size at birth, growth between 0 and 12 months, and food intake among a representative sample of Icelandic children. Dietary intake was assessed through monthly repeated 24-h records throughout the first 12 months and 3-day weighed records at 9 and 12 months of age. The children were weighed before and after breastfeeding. Measurement error in the dietary assessment was not considered.

Weight gain from birth to 12 months as a ratio of birth weight was positively related to BMI at the age of 6 years in both genders (β 2.9±1.0, *P*=0.008, and β 2.0±0.9, *P*=0.032 for boys and girls, respectively). Boys in the highest quartile of protein intake (E%) at the age of 9–12 months had significantly higher BMI at 6 years than the lowest and the second lowest quartiles (17.8±2.4 vs. 15.6±1.0 and 15.3±0.8, *P*=0.039 and *P*=0.01, respectively). Energy intake was not different between the groups. Together, weight gain at 0–12 months and protein intake at 9–12 months explained 50% of the variance in BMI among 6-year-old boys. The authors concluded that rapid growth during the first year of life is associated with increased BMI at the age of 6 years in both genders. In boys, high intake of protein in infancy could also contribute to childhood obesity. (Note: Bonferroni's correction for multiple tests was used when significant differences were found.)

Günther et al. (33), graded B, studied the AR among 7-year-olds in relation to protein intake between 12 and 24 months. Six repeated 3-day weighed records assessed dietary intake during the first 2 years, each covering 2 weekdays and 1 weekend day. In the present study, records from 12, 18 and 24 months were used. Measurement error in the dietary assessment was not considered, but all dietary variables were preadjusted for total energy intake using the residual method.

Girls in the highest tertile of habitual protein intake (E% and g/kg reference body weight [RBW]/day) had a significantly higher BMI SD score (BMI-SDS) at AR than those in the lower tertiles. In boys, there were no differences in BMI-SDS at AR between tertiles of habitual protein intake (E% or g/kg RBW/day). Boys in the lowest tertile of habitual protein intake (E%) tended to experience a later AR (*P*=0.07). But neither in girls nor in boys was age at AR significantly different between tertiles of habitual protein intake. The authors concluded that a higher habitual protein intake between the age of 12 and 24 months was associated with a higher BMI-SDS at AR in girls, but not in boys. There was no consistent relation between habitual protein intake in early childhood and timing of AR.

Günther et al. (34), graded A, analyzed the associations of different protein intakes during 6–24 months with BMI and percentage body fat (%BF) at 7 years, using data from the DONALD study in Germany. Dietary intake was assessed by 3-day weighed records during three consecutive days (2 weekdays and 1 weekend day) at 6, 12, 18, and 24 months. Breastfed infants were weighed before and after each feed. Goldberg's cut-off and Schofield equations were used to test the validity of the dietary assessment, and the residual model was used to adjust total protein intake for energy intake and sex. The median of these energy-adjusted protein intakes was used to distinguish different patterns of low and high protein intakes, and marked differences were observed between the low and high protein groups at 6 months (7–8 E% and 12 E%) and at 12 months (11–12 E% and 14–15 E%), respectively.

A consistently high protein intake at 12 and 18–24 months was independently related to a higher mean BMI SDS and %BF at 7 years and a higher risk of having a BMI or %BF above the 75th percentile. The analyses included adjustments for a large number of possible confounders, such as dietary factors (e.g. energy intake and breastfeeding) and parental characteristics (e.g. maternal overweight). Protein intake at 6 months was not associated with the outcomes. They conclude that their results suggest an association between high protein intakes during complementary feeding and the transition to the family diet with both a higher BMI and higher body fatness at 7 years of age.

Günther et al. (35), graded A, used data from the DONALD study to examine whether there may exist a critical period of protein intake for later obesity early in childhood and to analyze the relation between protein intake from different sources on BMI and %BF at 7 years of age. Dietary intake was assessed by 3-day weighed records during three consecutive days (2 weekdays and 1 weekend day) at 6, 12, 18, 24 months and yearly thereafter. Mean intake was calculated for the periods 18–24 months, 3–4 years, and 5–6 years. Breastfed infants were weighed before and after each feed, and 5% was added to the test weighing results to account for insensible water loss. Goldberg's cut-off and Schofield equations were used to test the validity of the dietary assessment, and the residual model was used to adjust total protein intake for energy intake and sex.

The ages of 12 months and 5–6 years were identified as critical periods at which higher total and animal, but not vegetable, protein intakes were positively related to body fatness at 7 years. Animal protein E% at 12 months was positively associated with BMI SDS at 7 years. The analyses were adjusted for several dietary and family characteristics, such as energy and fat intake and maternal overweight. When examining different sources of protein, dairy protein E% at 12 months, but not meat or cereal, showed a positive association with BMI SDS (*p*=0.02) and %BF at 7 years (*P*=0.07). The authors conclude that a higher intake of animal protein at 12 months, especially from dairy foods, might be associated with an unfavorable body composition at 7 years, and that the age of 5–6 years might represent another critical period of protein intake for later obesity risk.

Hoppe et al. (36), graded B, studied body size (BMI), body composition (percent body fat, %BF), and insulin-like growth factor I (sIGF-I) at 10 years of age among Danish children (*n*=142 at 9 months, 105 at 10 years) in relation to protein intake at 9 months and SUN at 9 months and 10 years. Dietary intake was assessed through 5-day weighed records at 9 months, including three weekdays and a weekend. (Note: It is not clear if measurement errors in the dietary recordings were considered. About one third of the children were breastfed at 9 months, but there is no information about whether breast milk intake was measured or if it is included in the nutrient calculations.) Adjustments were made for breastfeeding status at 9 months without any effect. Tanner stages were assessed at 10 years but not adjusted for in the analyses (96% of boys and 63% of girls had no sign of pubertal development).

In total, 7.8% of boys and 7.5% of girls were overweight, none were obese. SUN (mmol/l) at 9 months was a predictor for BMI and weight at 10 years. Protein intake (E%, g/day but not g/kg/day) at 9 months was a predictor for weight and height at 10 years. The associations remained when adjusting for parental body size, but were attenuated when adjusting for infant body size at 9 months.

Morgan et al. (37), graded B, examined body weight, length, and head circumference at 4, 8, 12, 16, 20, and 24 months in a cohort study of 144 infants. Dietary intake was measured through 7-day weighed food records at 4, 8, 12, 16, 20, and 24 months (meat intake calculated as average intake during 4–24 months). Meat intake from 4 to 12 months was positively and significantly related to weight gain. This association might be mediated via protein intake but was independent of energy, zinc, or iron intake. These findings remained after adjustment for potential confounding factors. There was no interaction between meat intake and breastfeeding on growth.

Öhlund et al. (4), graded B, did a follow-up at 4 years of age of healthy children (63 girls and 64 boys) previously followed prospectively from 6 to 18 months of age. Monthly 5-day weighed records measured dietary intake at 6–18 months and at 4 years. BMI at 6–18 months was a strong predictor of BMI at 4 years. Intake of protein in particular, and also of total energy and carbohydrates at 17/18 months and at 4 years, was positively associated with BMI at 4 years. Although BMI at 6–18 months was the strongest predictor of BMI at 4 years, in the final multivariate models of the child's BMI, high protein intake at 17–18 months and at 4 years, energy intake at 4 years, and the father's, but not the mother's, BMI were also independent contributing factors. One limitation of the study was that physical activity was not included as a factor when estimating energy requirements.

Scaglioni et al. (38), graded B, studied growth of healthy children (singleton birth, healthy parents) from birth to age 5 years. Dietary intake was assessed at 1 and 5 years-of-age through age-adjusted food frequency questionnaires (FFQs) complemented by a 24-h recall to standardize the usual serving size. Measurement error in the dietary assessment was not considered. The prevalence of overweight at the age of 5 years was strongly associated with parental overweight (*P*<0.0001), and overweight children had a higher intake of protein at the age of 1 year than non-overweight children (22E% vs. 20E%, *P*=0.024). In children born from overweight mothers, the prevalence of overweight at the age of 5 years tended to be higher in bottle-fed than in breastfed ones (62.5% vs. 23.3%, *P*=0.08). The authors concluded that parental overweight is a major risk factor for childhood overweight in the first years of life, but an early high protein intake may also influence the development of adiposity.

Skinner et al. (39), graded B, found in a prospective study of 70 children that their BMI at 8 years was negatively predicted by age of AR and positively predicted by their BMI at 2 years. Dietary intake was assessed through nine sets of non-consecutive 3-day data collections (2 days’ recordings and one 24-h recall). Measurement error in the dietary assessment was not considered. Longitudinal intake (between 2 and 8 years) of protein and fat (grams/day and E%) were positively related to BMI at 8 years, but mean carbohydrate intake over the same time period was negatively related to BMI at 8 years. A weakness, also mentioned by the authors, was that there were no observations to verify normal food intake nor was total energy expenditure measured by doubly-labelled water (DLW) methods.

Van Vught et al. (40), graded B, studied associations between intakes of total protein as well as the amino acids arginine (ARG) and lysine (LYS) in the habitual diets of normal weight and overweight 6-year-olds and subsequent linear growth, body fat, and fat free mass (FFM) at 9 years. Dietary intake was assessed through a 7-day estimated food record. Reporting of dietary intake was evaluated by comparing reported energy intake with estimated energy requirements. Children reporting an energy intake below BMR × 1.3 or above BMR × 2.0 were excluded.

High ARG intake was associated with linear growth among girls. Furthermore, in girls, fat mass index (FMI) had a stronger inverse association with high ARG intake, if it was combined with high LYS intake, instead of low LYS intake (*P*=0.03). No associations were found in boys although the change was in the same direction as for the girls. The authors conclude that in pre-pubertal girls, linear growth may be influenced by habitual ARG intake, and body fat gain may be relatively prevented over time by the intake of the amino acids ARG and LYS.

In another study, Van Vught et al. (41), graded B, studied associations between intakes of protein, ARG, and LYS among 8–10-year-olds and subsequent 6-year change in body composition (fat-free mass index [FFMI] and FMI). Dietary intake was assessed through one 24-h recall.

Note: Measurement errors were discussed but not measured. The authors suggest that among lean girls, high protein intakes at 8–10 years may decrease subsequent body fat gain and increase fat free mass gain depending on the available amounts and combinations of ARG and LYS.

#### Cross-sectional studies

Budek et al. (14), graded B, studied concentrations of serum insulin-like growth factors (sIGF-I) in relation to intake of total dairy and meat protein in pre-pubertal 8-year-old boys. Dietary intake was assessed using a 3-day weighed food record (2 weekdays and 1 weekend day). Measurement errors in the dietary recordings were not considered, but all dietary variables were preadjusted for total energy intake using the residual method. Free sIGF-I was positively associated with total protein (*p*<0.01) and dairy protein (*p*=0.06) but not with meat protein. (Note: Meat protein included red meat, poultry, and fish, but not pork. Plant protein was estimated from the difference between total protein intake and intake from dairy, meat, and egg.) Measurement errors in the dietary recordings were not considered, except for mentioning that dietary assessment in children is difficult.

Hoppe et al. (13), graded B, examined associations between protein intake, sIGF-I concentrations, and height in a cross-sectional study of 2.5-year-old healthy children (n = 90) in Denmark. Dietary intake was reported through a 7-day estimated food record with pre-coded response categories. Amounts were given as household measures or as standard portion sizes estimated from a picture booklet. Measurement errors in the dietary recordings were not considered. The 10th, 50th, and 90th percentiles of protein intake were 2.4, 2.9, and 4.0 g/kg/day, respectively; 63% was animal protein.

In multiple linear regressions with adjustment for sex and weight, height and sIGF-I concentration were positively associated with each other as well as with intakes of animal protein (g/day) and milk but not with those of vegetable protein or meat. The authors concluded that milk intake was positively associated with sIGF-I concentrations and height. An increase in milk intake from 200 to 600 ml/day corresponded to a 30% increase in circulating sIGF-I. The authors suggest that milk compounds have a stimulating effect on sIGF-I concentrations and, thereby, on growth.

Kourlaba et al. (42), graded B, studied interaction effects between energy and macronutrient intakes and angiotensin-converting enzyme 1 (ACE) I/D polymorphism on adiposity-related phenotypes among 1–5-year-olds in the Greek Genesis study. Dietary intake was assessed through 3-day records (2 weekdays and 1 weekend day) combining weighed food records (by staff during nursery hours) and 24-h recall. Measurement errors in the dietary recordings were discussed but not measured.

Significant interactions were found between the *ACE* I/D polymorphism and protein intake on BMI and being overweight. Stratified analyses revealed that protein intake was associated with BMI and being overweight only among carriers of the D-allele (i.e. DD or ID genotypes). Protein intake was higher among those ‘at risk of being overweight’ or ‘overweight’ compared with their normal-weight counterparts. The authors conclude that the results suggest that the *ACE* I/D polymorphism may act as a modifying factor in the response of adiposity-related phenotypes to diet and that further research is required to confirm their findings.

Maillard et al. (43), graded B, studied growth in relation to dietary intake among 5–11-year-old non-obese pre-pubertal children. Dietary intake was assessed through 1-day records, that is, one out-of-school-weekday. Intake data were validated with Schofield's equation and Goldberg's & Black's cut-off. They found that associations between adiposity and protein intake as E% or energy intake were non-significant. All adiposity indices (except waist girth and BMI) were significantly and inversely associated in girls with energy intake from carbohydrates (%EIC, all *P*-values <0.02), and positively with energy intake from fat (%EIF, all *P*-values <0.05). Similar but non-significant trends were observed in boys. The relationships were not linear, and thresholds close to current dietary recommendations were highlighted.

Manios et al. (44), graded B, studied anthropometrical indices in relation to nutrient intake (based on 3-day food records; 2 weekdays, 1 weekend day) among children aged 1–5 years. Intake data were validated with Schofield's equation and Goldberg's & Black's cut-off. Bonferroni correction was used. For both fat and carbohydrate, a substantial percentage of toddlers and preschoolers had usual intakes outside the acceptable macronutrient distribution range, whereas protein was less than this range.

No statistically significant differences were seen in protein E% between children with normal weight (17.1±1.6), at risk of overweight (17.1±1.5), or overweight (17.1±1.5). However, children ‘at risk of overweight’ and ‘overweight’ had higher intake of total energy, protein (g/day), and fat (g/day) compared with their normal-weight counterparts (*p*<0.001 for all), whereas no differences were found for micronutrient intakes.

#### Complementary search

Closa-Monasterolo et al. (45), graded B, aimed to investigate whether sex modulates the responses of relevant biochemical parameters and growth to different protein intakes early in life. In randomized controlled trials (RCT) in five European countries [the same participants as in (29)], formula-fed infants were assigned to receive formula with lower or higher protein content (cow-milk-based infant formula with 1.77 or 2.9 g protein/100 kcal) from a median age of 14 days. The protein content represented approximately the lowest and highest acceptable levels in the range given in the EU directives from 1991.

Protein (g/day) and energy intake (kcal/day) was assessed by 3-day weighed records during three consecutive days (2 weekdays and 1 weekend day) at 3 and 6 months. Measurement errors in the dietary recordings were not considered. Outcomes (e.g. sIGF-I axis parameters, weight, length, BMI, and leptin) were measured at 6 months. The authors conclude that their findings indicate that the endocrine response to a high protein diet early in life may be modulated by sex. The sIGF-I axis of female infants showed a stronger response to the intervention, but there was no enhanced effect on growth.

#### Conclusion

##### BMI/growth

Thirteen studies (one CT, nine cohort, three cross-sectional) found an association between higher protein intake in different age groups and increased growth/higher BMI; of the 13 studies, 1 study concluded that the combinations of amino acids in infant formula matters, while 4 studies (two CT, one cohort, and one cross-sectional) saw no effect although one of them saw a positive association with the intake of certain amino acids (Table 2).

In an intervention study, Koletzko et al. (29) found that a higher protein content of infant formula was associated with higher weight in the first 2 years of life but had no effect on length compared with infants fed a low-protein formula. Compared with breastfed children, those fed high-protein formula had significantly higher z-scores for weight, length, weight-for-length, and BMI at 24 months.

Intake during the first year and outcomes between 5 and 10 years of age was studied in three cohort studies. Gunnarsdottir et al. (3) found an association between high intake of protein in infancy and increased risk for childhood obesity at 6 years only among boys, while rapid growth during the first year of life was associated with increased BMI at the age of 6 years in both genders. Scaglioni et al. (38) found a positive association between protein intake at 1 year and the risk of overweight at 5 years, although they conclude that parental overweight is a major risk factor for childhood overweight in the first years of life. Hoppe et al. (36) found that protein intake (E% and g/day, but not g/kg/day) at 9 months was a predictor for weight and height at 10 years.

Three cohort studies looked at protein intake in early childhood and outcomes at 4–10 years. Öhlund et al. (4) found that BMI at 6–18 months was the strongest predictor of BMI at 4 years, but protein intake at 17–18 months and at 4 years, energy intake at 4 years and the father's, but not the mother's, BMI were also independent contributing factors. Günther et al. (34) found that consistent high protein intake at 12 and 18–24 months, but not 6 months, was positively related to a higher mean BMI SDS (SD score) at 7 years and a higher risk of having a BMI above the 75th percentile. Skinner et al. (39) found that mean protein and fat intakes recorded between 2 and 8 years were positive predictors of BMI at 8 years.

Four studies, (one CT, two cohort and one cross-sectional), focused on the effects of different kinds of amino acids and protein. In an intervention study, Sandström et al. (31) found that weight gains in groups fed α-lactalbumin-enriched formula were similar to that of the breastfed infants, while infants fed the standard formula gained significantly more weight than did the breastfed infants. Morgan et al. (37) concluded that meat intake from 4 to 12 months was positively and significantly related to weight gain during the first 2 years; further analysis suggested this association might be mediated via protein intake but was independent of energy, zinc or iron intake. In contrast, Günther et al. (35) found that dairy protein E% at 12 months, but not meat or cereal, showed a positive association with BMI SDS a 7 years. In a cross-sectional study, Hoppe et al. (13) showed that height (cm) at 2.5 years was positively associated with intakes (g/day) of animal protein and milk, but not with intakes of vegetable protein and meat.

Two cross-sectional studies found that the intake of protein (42), and energy, protein, and fat (44), was higher among those ‘at risk of being overweight’ or ‘overweight’ compared with their normal-weight counterparts.

Four studies, that is, two CT, one cohort and one cross-sectional, found no association between protein intake and growth. The cross-sectional study by Maillard et al. (43) found no effect of protein intake on growth (several different indices) among 5 to 11-year-old pre-pubertal children, but inverse association with energy intake from carbohydrates and positive associations with energy intake from fat. These associations were significant for girls and similar but non-significant trends were observed in boys. The intervention study by Larnkjær et al. (30) found no effect of milk type (whole milk or infant formula) on growth between 9 and 12 months. However, drop-outs (17% of whole milk and 6% of infant formula) were 1.6 cm shorter at 9 months, which might have affected the results. The second intervention study, by Räihä et al. (5), found no difference in BMI or weight- and length-gains between four feeding groups (breastfed vs. formula-fed with different protein content and ratio whey/casein) during the first 120 days. One study from Van Vught (40) found no association between protein intake and growth for either sex although they found a positive association between the amino acid ARG and linear growth. In addition, the study found in the complementary search (45) found no enhanced effect on growth from increased protein intake in infancy.

##### Adiposity rebound (AR)

Two cohort studies found no association between protein intake and timing of AR. Dorosty et al. (32) found no evidence for an association between protein intake at 18 months, or any other dietary variable, and timing of the AR. Günther et al. (33) did not find any consistent relation between habitual protein intake in early childhood and timing of AR either, but concluded that a higher habitual protein intake between the age of 12 and 24 months was associated with a higher BMI-SDS at AR in girls, but not in boys.

##### Body composition

Two A-graded cohort studies from the same group (34, 35) found a positive association between protein intake and %BF, while two studies from another group (40, 41) found inverse associations between protein intake and FMI among lean girls. Günther et al. (34) found that consistently high protein intake at the ages of 12 and 18–24 months, but not 6 months, was positively related to a higher mean %BF at 7 years and a higher risk of having a %BF above the 75th percentile. In another study, they identified the ages of 12 months and 5–6 years as critical periods at which higher total and animal (especially from dairy), but not vegetable, protein intakes were positively related to body fatness at 7 years (35).

Van Vught et al. (40) conclude that in pre-pubertal girls, body fat gain may be prevented over time by the intake of the ARG and LYS. In another study (41), among lean girls in 3rd–9th grade, they found inverse associations between intake of protein as well as of ARG and LYS and change in FMI.

Based on a cross-sectional study, Kourlaba et al. (42) suggest that responses to diet of different adiposity-related phenotypes may be modified by *ACE* I/D polymorphism (but they also state that further research is required to confirm their findings).

##### sIGF-I

Five studies (four from the same group, but with different children) found positive associations between milk intake and concentrations of sIGF-I. In a cross-sectional study, Hoppe et al. (13) found that milk intake was positively associated with sIGF-I concentrations and height among 2.5-year-old boys and girls. The same group conducted a short 7-day intervention study among 8-year-old boys (*n*=24) and found that high intake of milk, and not meat, increased concentrations of sIGF-I and sIGF-I/sIGFBP-3 (28). In another cross-sectional study among 8-year-old boys (14), the same group found that free sIGF-I was positively associated with total protein (*p*<0.01) and dairy protein (*p*=0.06) but not with meat protein. In a later, and larger 7-day intervention study on boys (*n*=57) (27), they concluded that casein stimulates circulating sIGF-I and that both milk protein fractions (whey and casein) seemed to be important, but different, in the growth-stimulating effect of milk. In a randomized trial between 9 and 12 months of age, Larnkjær et al. (30) found that whole milk, but not infant formula, increased sIGF-I in boys, but not in girls. They also found that high protein intake (E%) was positively associated with sIGF-I concentration in both boys and girls. The results found in the complementary search (45) indicated that the endocrine response to a high protein diet early in life may be modulated by sex.

Based on the above, we conclude that evidence is convincing (grade 1) that higher protein intake in infancy and early childhood is associated with increased growth and higher BMI in childhood. There is limited-suggestive evidence (grade 3) that the intake of animal protein, especially from dairy, has a stronger association with growth than vegetable protein has. The association found between higher intake of milk and increased levels of sIGF-I strengthens this finding.

There is limited-inconclusive evidence (grade 4) that protein intake is related to timing of AR. Due to a scarcity of strong studies, there is also limited-inconclusive evidence (grade 4) that protein intake in later childhood is associated with later BMI. The evidence is also limited-inconclusive (grade 4) (due to the two A-graded studies not being independent) that there is an association between higher protein intake in early childhood and later body fat increases. There might also be different effects depending on BMI, phenotypes, and gender. This conclusion is supported by the randomized trial on endocrine responses to high protein diets in infancy found in the complementary search (45), although it is not enough to change the grading of the evidence.

### Bone health

Table 4 shows a summary of studies with outcome bone health (details are provided in appendix 4). In total, six papers, all graded B, were chosen in the systematic review process to be evaluated for the evidence of an association between protein intake and bone health. Of those six, one was a clinical trial, two were prospective cohort studies, and three were cross-sectional studies. Four studies came from the same research group. (One additional study was graded C and therefore excluded).

**Table 4:** Protein intake and outcome bone health (1 clinical trial, 2 cohort, 3 cross-sectional)

Author, year (ref no.) Country Study design (study name if applicable)	No. of participants	Exposure (incl age)	Outcome (incl age)	Effect/association	Study quality comments
Alexy, 2005 (47) Germany Prospective cohort, subgroup DONALD Study	229	Potential renal acid load (PRAL) calculated from dietary protein, P, Mg, K. Exposure to protein, PRAL and Ca = input	Proximal forearm bone variables (cross-sectional data 1 measurement) 6–18 years	Protein intake (g/day) was positively associated with all bone variables (explained 3–6% of variation in bone indexes). PRAL was negatively associated with cortical area (*p*=0.0075) and bone mineral content (*p*=0.0055). Explained 2% of variation for both. (Muscle area accounted for 24–36%, *p*<0.001) Ca intake non-sign for all bone variables.	By chance findings not considered 38% had non-valid records (Goldberg and Schofield) and were excluded. No details given as to level of underreporting among those remaining in study. Mean energy intake ca 74% (pre-pubescent girls) −84% (pre-pubescent boys) of recommendation for age.
Bounds 2005, (48) USA Prospective cohort	52 (25 M, 27 F)	Children's dietary intake, height, weight, and level of sedentary activity were assessed as part of a longitudinal study from ages 2 months to 8 years	Total BMC (bone mineral content, g) and BMD (bone mineral density, g/cm^2^) at age 8 years.	Factors positively related to children's BMC at age 8 years included longitudinal intakes (ages 2 to 8 years) of protein, phosphorus, vitamin K, magnesium, zinc, energy, and iron; height; weight; and age (*p ≤* 0.05). Factors positively related to children's BMD at age 8 years included longitudinal intakes of protein and magnesium (*p*≤0.05). Female sex was negatively associated with BMC and BMD at age 8 years (*p*≤0.05). Children's bone mineral indexes at ages 6 and 8 years were strongly correlated (*r*=0.86, *p*<0.0001 for BMC; *r*=0.92, *p*<0.0001 for BMD.	B Measurement errors not considered (but it is stated that throughout this longitudinal study, mothers received training from RDs on estimating portion sizes and keeping precise food records.and the RDs reviewed food records for completeness and accuracy at each interview), no power calculation
Budek, 2007, (49) Denmark Cross-sectional	109 (46 M, 63 F)	Milk and meat protein intake at 17 years. The aim was to test the hypotheses that total protein intake is positively associated with bone mass, and that milk and meat protein intake isdifferently associated with bone mass in adolescents	bone mineral content (BMC) at 17 years	The mean total protein intake (∼1.2 g/kg) was modestly higher than that recommended. Total and milk (∼0.3 g/kg) protein intake, but not meat protein intake (∼0.4 g/kg), was positively associated with size-adjusted BMC (*p*≤0.05). The positive association between milk protein intake and size-adjusted BMC remained significant after correction for energy, calcium, and physical activity (*p*≤0.01) and did not seem to be mediated via current serum IGF-I. None of the analyzed protein sources was significantly associated with size-adjusted BA. Conclusions Our results suggest that some components of milk protein may promote bone mineralization. Further studies are needed to elucidate this phenomenon.	B No power calculation, no adjustment for mis/underreporting, girls reported energy intake in lower range but OK
Budek, 2007, (14) Denmark, Cross-sectional	81 boys (out of 96 eligible boys)	Intake of total, dairy and meat protein	Concentrations of sIGF-I and markers for bone-turnover (serum osteocalcin (sOC), bone-specific alkaline phosphatase (sBAP) and C-terminal telopeptides of type l collagen (sCTX) measured by immunoassay)	Dairy protein was negatively associated with sOC (*p*=0.05) but not significantly associated with sBAP and sCTX. Further analyses showed that dairy protein decreased (*p*=0.05) sOC at a high meat protein intake (>0.8 g/kg), whereas meat protein increased (*p*=0.03) sOC at a low dairy protein intake (<0.4 g/kg). Total and meat protein intake was positively associated with sBAP (*p*≤0.04) but not significantly associated with sOC and sCTX. Free sIGF-I was positively associated with total (*p*<0.01) and dairy (*p*=0.06) protein but not with meat protein. Our results indicate that dairy and meat protein may exhibit a distinct regulatory effect on different markers for bone turnover. Future studies should focus on differential effects of dairy and meat protein on bone health during growth.	B Results not adjusted for mis/underreporting, no power calculation. In discussion: ‘Meat protein intake was estimated from the intake of red meat, poultry and fish.’ Why is fish included? What about pork? Egg? ‘Plant protein intake was estimated from the difference between total protein intake and dairy, meat and egg protein intake.’
Budek, 2007 (46) Denmark Short-term (7 days) intervention study	24 boys	Protein intakeOne group: milk (1.5 l/day) and the other group: meat (250 g/day) Both containing about 53 g of protein. Otherwise, habitual diet.	Markers for bone-turnover: Serum osteocalcin (sOC), bone-specific alkaline phosphatase (sBAP) and C-terminal telopeptides of type l collagen (sCTX) measured by immunoassay 8 years	Baseline sOC, sBAP and sCTX were not sign different between the groups. After 7 days, the average protein intake increased in both groups by 47.5 g; the milk group had higher (*p*<0.0001) calcium intake; sOC and sCTX decreased (*p*<0.04) in the milk group (−30.9%; −18.7%, respectively) compared with the meat group (+6.4%;−1.0%, respectively) and sBAP decreased (*p*=0.06) both in the milk (−3.9%) and the meat group (−7.5%).The milk group had significantly higher calcium intake compared with the meat group and this could also affect the decline observed in bone turnover in the milk group. Whether this decline promotes higher bone mineral accretion during growth needs to be further studied according to the authors.	B No power calculationCompliance is not reported. It is stated that protein intake is increased but nothing about how much of the additional 15 dl of milk/250 g of meat was actually taken, and very little about how their habitual diet changed. (1.5 l skim milk = 2.3 MJ and +23% of mean energy intake, 250 g meat = ca 1.6 MJ and +16% of mean energy intake. The average increase was total + 13% in the milk group and +3% in the meat group).
Hoppe, 2000 (50) Denmark Cross sectional	105	Usual dietary intake at 10 years Energy, protein and other nutrients	Whole body bone mineral content (BMC, g) and bone size expressed as anterior-posterior projected bone area (BA, cm^2^)	In bivariate analyses, BMC and BA were positively correlated with height (*p*<0.001) and weight (*p*<0.001) and with intakes of energy (*p*<0.005) and several nutrients. In multivariate analyses, size-adjusted BMC was positively associated with calcium intake (*p*=0.02), and size-adjusted BA was positively associated with dietary protein (*p*=0.003), and negatively associated with intakes of sodium (*p*=0.048) and phosphorus (*p*=0.01).	B Energy adjustment not done (fat, protein and carbs are reported as g/day) No power calculation

#### Clinical trials

Budek et al. (46), graded B, compared the short-term (7 days) effect of high milk and high meat intake on bone turnover during pre-puberty among 24 Danish boys aged 8 years assigned to either 1.5 l milk per day or 250 g meat per day (both containing 53 g protein) given together with the habitual diet for 7 days. The participants all had a habitual milk intake of at least 500 ml/day before the start of the study. Dietary intake was assessed using two repeated 3-day weighed food records (2 weekdays and 1 weekend day); the first kept before the intervention (days −3 to 0) and the second at the end of the intervention (days 5 to 7). Measurement errors in the dietary recordings were not considered, but the importance of maintaining usual dietary intake was emphasized to the families.

There were no significant differences between the groups’ intake of total energy (MJ/day) or protein (g/day, g/kg/day) neither before nor after the intervention. In the milk group, the proportion of energy from carbohydrates increased slightly and the proportion from fat decreased with an average 8%-units (from 34.6 E% to 26.7 E%), while the meat group decreased the proportion of energy from carbohydrates with on average 10%-units (from 56.6 to 46.8 E%) and increased the proportion from fat with about 2%-units.

At equal protein intake, milk, but not meat, decreased markers for bone formation and resorption after 7 days. The authors suggest that this effect was more likely due to some milk-derived compounds, rather than to the total protein intake. The milk group also had significantly higher calcium intake compared with the meat group and this could affect the decline observed in bone turnover in the milk group. Whether the decline in bone turnover markers promotes higher bone mineral accretion during growth needs to be further studied according to the authors.

#### Prospective cohort studies

Alexy et al. (47), graded B, examined the association of long-term protein intake and dietary potential renal acid load (PRAL) with bone variables in a subgroup of 229 children and adolescents (6–18 years) in the DONALD study. Proximal forearm was measured once (cross-sectionally) at 6–18 years of age, and dietary intake was measured through yearly 3-day weighed records over the 4-year period prior to bone measurements. Goldberg's cut-off and Schofield equations were used to test the validity of the dietary assessment (38% had non-valid records and were excluded). Collinearity diagnostics detected no evidence for collinearity between the dietary factors.

Reported protein intake measured as g/day was significantly lower in prepubescent boys and girls compared with pubescent boys and girls, while the intake measured as g/kg/day was higher in the younger children. Long-term protein intake was positively associated with all bone variables (periosteal circumference, cortical area (CA), bone mineral content (BMC), and polar strength strain index at the proximal diaphyseal radius) in these healthy children and adolescents, also after adjustment for age, sex, and energy intake, and control for forearm muscularity, BMI, growth velocity, and pubertal development. Children with a higher dietary PRAL (i.e. indicating an inadequate intake of alkalizing minerals from vegetables and fruit) had significantly less CA and BMC. Calcium intake had no significant effect on any bone variable. The authors emphasize the importance of evaluating the whole diet when studying dietary influence on bone health.

Bounds et al. (48), graded B, studied total BMC (g) and bone mineral density (BMD, g/cm^2^) measured by dual energy X-ray absorptiometry (DEXA) scan at 8 years among 52 children (25 boys, 27 girls) in relation to an averaged longitudinal nutrient intake between 2 and 8 years (measured as average of 3 days [2 days with estimated food records and one 24-h recall] times nine occasions). Measurement errors in the dietary recordings were not considered, but it is stated that the parents received training from registered dieticians. Mothers’ BMC was also assessed by DEXA and the time children spent in sedentary activities was assessed through a questionnaire at 6 and 7 years of age. Multivariate models predicting children's total BMC and BMD at 8 years of age were developed using stepwise regression procedures adding all potentially independent variables.

Factors positively related to children's BMC at age 8 years in the final models included longitudinal intakes (ages 2 to 8 years) of protein, phosphorus, vitamin K, magnesium, zinc, energy, and iron; height; weight; and age. Factors positively related to children's BMD at age 8 years included longitudinal intakes of protein and magnesium. Female sex was negatively associated with both BMC and BMD at age 8 years in contrast with previous studies, that have been unable to find sex differences in pre-pubertal children. Children's bone mineral indexes at ages 6 and 8 years were strongly correlated (*r*=0.86, *P*<0.0001 for BMC; *r*=0.92, *P*<0.0001 for BMD).

#### Cross-sectional studies

Budek et al. (49), graded B, studied BMC and bone area (BA) determined by DEXA, in relation to milk and meat protein intake at 17 years. Dietary intake was reported through a 7-day estimated food record with pre-coded response categories, supplemented with open-ended alternatives. Physical activity level was recorded using a 24-h recall questionnaire, assessing total hours per day spent at four different activity levels. Time spent on high activity level was used in the statistical analyses.

Total protein intake (∼1.2 g/kg) and milk protein intake (∼0.3 g/kg), but not meat protein intake (∼0.4 g/kg), was positively associated with size-adjusted BMC. The positive association between milk protein intake and size-adjusted BMC remained significant after correction for energy, calcium, and physical activity and did not seem to be mediated via current serum sIGF-I. None of the analyzed protein sources were significantly associated with size-adjusted BA. The authors suggest that some components of milk protein may promote bone mineralization.

Note: Measurement errors in the dietary recordings were not considered, except for mentioning in the discussion that the dietary assessment might include bias due to inadequate recording (however, no details are given). Pubertal stages were not considered.

Budek et al. (14), graded B, also studied concentrations of sIGF-I and markers for bone-turnover (serum osteocalcin [sOC], bone-specific alkaline phosphatase [sBAP], and C-terminal telopeptides of type l collagen [sCTX]) in relation to intake of total dairy and meat protein in pre-pubertal 8-year-old boys. Dietary intake was assessed using a 3-day weighed food record (2 weekdays and 1 weekend day). Measurement errors in the dietary recordings were not considered, except for mentioning that dietary assessment in children is difficult. All dietary variables were preadjusted for total energy intake using the residual method. Note: Meat protein included red meat, poultry, and fish, but not pork. Plant protein was estimated from the difference between total protein intake and intake from dairy, meat, and egg.

Dairy protein was negatively associated with sOC but not significantly associated with sBAP and sCTX. Further analyses showed that dairy protein decreased sOC at a high meat protein intake (>0.8 g/kg), whereas meat protein increased sOC at a low dairy protein intake (<0.4 g/kg). Total and meat protein intake was positively associated with sBAP but not significantly associated with sOC and sCTX. Free sIGF-I was positively associated with total and dairy (*P*=0.06) protein but not with meat protein. The authors suggest that their results indicate that dairy and meat protein may exhibit a distinct regulatory effect on different markers for bone turnover.

Hoppe et al. (50), graded B, studied associations between dietary factors and whole body BMC and BA determined by DEXA in a cross-sectional analysis of 105 10-year-old Danish children. Dietary intake was reported through a 7-day estimated food record with pre-coded response categories, supplemented with open-ended alternatives. Goldberg's cutoff was used to test the validity of the dietary assessment, and no implausibly low energy intakes were reported.

The mean intakes of calcium, protein, phosphorus, and sodium were 1226 mg, 78 g, 1523 mg, and 3.3 g, respectively, all considerably higher than the Nordic recommendations. In bivariate analyses, BMC and BA were positively correlated with height and weight and with intakes of energy and several nutrients. In multivariate analyses, size-adjusted BMC was positively associated with calcium intake, and size-adjusted BA was positively associated with dietary protein, and negatively associated with intakes of sodium and phosphorus. Inclusion of pubertal stages in the multiple linear regressions did not alter the outcome.

#### Complementary search

Libuda et al. (51), graded B, examined as part of the DONALD cohort study, the effects of nutrients (e.g. protein, calcium, and vitamin D) on bone parameters and compared their effects sizes with those of two known predictors of bone development: bone-related muscle mass and androgen levels. Long-term nutrient intakes were assessed by 3-day weighed dietary records and calculated as the mean of each dietary record in the 4 years before measurement of bone and muscle variables, for example, diaphyseal BMC, CA, and muscle area.

Of all considered nutrients, only protein showed a trend for an association with BMC (β= +0.11; *P*=0.073) and CA (β = + 0.11; *P*=0.056). None of the other dietary variables was associated with the bone parameters. The size of the bone anabolic effect of protein was partly comparable with that of androstenediol. The authors conclude that their results suggest that bone-related muscle area has the strongest effect on bone status of healthy prepubertal children followed by the sex steroid androstenediol and protein intake, which was found to be the strongest dietary predictor of diaphyseal bone.

Mark et al. (52), graded B, is a continued analysis of the data from the intervention study by Hoppe et al. (27), studying the effects of milk-derived compounds on bone formation and resorption during growth in a 7-day randomized double-blind study among 8-year-old Danish boys with a habitual milk intake not exceeding 500 ml/day. Markers for bone-turnover (serum osteocalcin [s-OC], bone-specific alkaline phosphatase [s-BAP], and C-terminal telopeptides of type l collagen [s-CTX]) were measured. The boys received one of four milk drinks, that is, whey protein with low or high content of minerals or casein protein with low or high content of minerals. The amount of whey and casein was identical to the content in 1.5 l of milk. Dietary intake was assessed using two repeated 3-day weighed food records (2 weekdays and 1 weekend day); the first kept before the intervention (days −3 to 0) and the second at the end of the intervention (days 5 to 7). Measurement errors in the dietary recordings were not considered, but the importance of maintaining usual dietary intake was emphasized to the families.

The intake of milk drinks containing whey protein increased sOC at the low content of milk minerals, whereas it decreased sOC at the high content of milk minerals (*P*<0.05). In contrast, the intake of milk drinks with casein protein increased sOC both at low and high content of milk minerals. They concluded that whey and casein differently affected sOC in 8-year-old boys depending on the content of milk minerals, but that it did not seem to affect other markers for bone turnover.

#### Conclusion

Five studies, (14, 47–50), report a positive association between total protein intake and BMC and/or other bone variables in childhood and adolescence. The short-term intervention study by Budek et al. (46) conclude that at equal protein intake, milk, but not meat, decreased markers for bone formation and resorption after 7 days. Three of the six studies were performed in 8-year-olds, one in 10-year-olds, and one in 6–18-year-olds, both adjusting for pubertal stage, and one in 17-year-olds not adjusting for pubertal stage. Only two of the six studies considered the validity of the dietary assessment.

Based on the above, we conclude that evidence is limited-suggestive (grade 3) for a positive association between total protein intake and BMC and/or other bone variables in childhood and adolescence. The two papers found in the complementary search support this conclusion, although we do not consider it enough to change the evidence grading.

### Puberty

Table 5 shows a summary of studies with outcome puberty (details are provided in appendix 5). In total, four papers were chosen in the systematic review process to be evaluated for the evidence of an association between protein intake and puberty. All four papers were prospective cohort studies, of which three (one A-graded and two B-graded) originated from the same study (DONALD). The fourth study was graded B.

**Table 5:** Protein intake and outcome puberty (4 cohort studies)

Author, year (ref no.) Country Study design (study name if applicable)	No. of participants	Exposure (incl age)	Outcome (incl age)	Effect/association	Study quality comments
Berkey, 2000 (53) USA Prospective cohort 1930s–1940s	67 girls followed from utero to 18 years	Dietary intake (kcal/day, animal protein g/day, vegetable protein g/day, total fat g/day) BMI averaged over multiyear periods (1–2, 3–5, 6–8 years + 1 and 2 years before peak growth)	Age at menarche, age at peak height growth velocity, peak growth velocity. Weight, height semi-annually 0–11 years, annually 11–18 years	For peak growth velocity the same three factors emerged in all age periods; more calories, more animal protein and lower BMI were consistently associated with higher peak growth velocity (factors closer to puberty more important). Timing of puberty was predicted by protein intake and height. Higher animal protein (energy adjusted) intake and less vegetable protein at 3–5 years had earlier menarche (+1 SD animal protein intake gave 0.63 year earlier menarche than −1 SD, and peak height growth was +0.6 cm/year). Higher dietary fat intake at 1–2 years associated with earlier peak growth (+1 SD fat intake gave 0.63 year earlier peak growth velocity than −1 SD) and higher calorie intake at 1–2 years gave higher peak height velocity (+1 SD calorie intake gave +1.11 cm/year than −1 SD) and higher animal protein intake at 6–8 years had earlier peak growth. Later age at menarche associated with lower age at peak growth (*r*=0.81, *p*<0.05) and lower peak growth velocity (*r*=−0.41, *p*<0.05).	B Drop-out rate high (43%)
Günther, 2010 (54) Germany DONALD Prospective cohort Study	112 children (92 had data on age at menarche/voice break)	Protein at 12 months, 18–24 months, 3–4 years, 5–6 years(total protein, animal and vegetable protein, and dairy, meat and cereal protein intake) + Energy intake, carbohydrates, fiber and fat intake	Timing of puberty; 1) Age at take-off of pubertal growth spurt (ATO) 2) Age at peak height velocity 3) Menarche/voice break	Higher animal protein intake (E%) at 5–6 years was associated with earlier puberty. Highest tertile of intake had ATO 0.6 year earlier than the lowest tertile (*p*=0.048). Similar tendency at 3–4 years.Vegetable protein intake (E%) associated with a later ATO. Protein intake from cow milk and dairy products at age 5–6 years (but not meat) was associated with an earlier ATO [mean ATO, 95% CI; tertile 1: 9.5, 9.2–9.8; tertile 2: 9.5, 9.3–9.8; tertile 3: 9.1, 8.8–9.4 year; *p*-trend = 0.04]. Children with higher animal protein intake (E%) at 3–4 and 5–6 years had earlier APHV [3–4 years: tertile 1: 12.5, 12.2–12.9; tertile 3: 12.0, 11.7–12.3 years; *p*<0.05], [5–6 years: tertile 1: 12.8, 12.5–13.1; tertile 3: 12.0, 11.7–12.3 years; *p*<0.05], while those with high vegetable protein intake had later APHV [3–4 years: tertile 1: 12.1, 11.8–12.5; tertile 3: 12.6, 12.3–13.0 years; *p*-trend = 0.02], [5–6 years: tertile 1: 12.2, 11.8–13.6; tertile 3: 12.6, 12.2–13.0 years; *p*=0.04]. Higher animal protein intake (especially milk) at 3–4 years tended (*p*=0.06) to be (and at 5–6 years was (*p*=0.02), associated to earlier menarche/voice break and later for high vegetable protein intake (0.02 and 0.03 respectively). Adjustment for confounders did not change all these associations.	A
Remer, 2010 (55) Germany. DONALD Prospective cohort Study	109	Energy and animal protein intake at 1 and 2 years before puberty onset	Timing of puberty; 1) Age at take-off of pubertal growth spurt (ATO) 2) Age at peak height velocity (APHV) 3) Menarche/voice, 4) Tanner stage 2 for breast (girls) and genital (boys) development	Higher adrenarchal C19 steroids predicted earlier ages at Tanner stage 2 for pubic hair (*p <* 0.0001) and B2-G2 (*p*=0.009) as well as a shorter pubertal growth acceleration period (*p*=0.001), independently of animal protein intake. Children with a higher Adrenal Androgen (AA) secretion had a 1.5-year earlier beginning of pubarche and a 0.8-year earlier beginning of B2-G2 than those with a lower AA excretion. Furthermore, animal protein intake was independently negatively associated with ATO and APHV (*p*<0.05 each) and tended to be negatively associated with age at menarche/voice break (*p*= 0.07).	B. No information on physical activity, statistical power, follow-up period and time-exposure variable not totally clear.
Shi, 2009, (56) Germany, DONALD Prospective cohort Study	137	Anthropometry; Nutrient intake including protein, and also glycemic index and glycemic load; Body composition such as fat mass, fat-free mass	Adrenachal androgen status (AA)	AA is depending on FM (5%, *p*<0.0001) and protein intake (1%, *p*<0.05).	B. No information on physical activity, statistical power, time of baseline or exposure variable not totally clear. Note: refer to previous papers for validation or exact description of dietary methods

#### Prospective cohort studies

Berkey et al. (53), graded B, studied the relation of childhood diet and body size to menarche and adolescent growth in a longitudinal study of 67 Caucasian girls in Boston, United States. Data were collected prospectively from birth to 18 years during the 1930s–40s. Dietary intake was assessed through dietary history interviews every 6 months up to 11 years of age, and annually thereafter. The reliability of total protein intake was estimated to be 71%, and the validity was tested by correlation between reported daily protein intake and the child's rate of growth of muscle in the lower leg.

They found that age at menarche, age at peak height growth velocity and peak height growth velocity were all associated with diet and body size earlier in childhood. For peak growth velocity, the same three factors emerged in all age periods, that is, more calories, more animal protein, and lower BMI were consistently associated with higher peak growth velocity (factors closer to puberty more important). Timing of puberty (age at menarche and age at peak growth velocity) was predicted by protein intake and height. Girls with higher animal protein (energy adjusted) intake and less vegetable protein at 3–5 years had earlier menarche. Higher dietary fat intake at 1–2 years was associated with earlier peak growth, and higher calorie intake at 1–2 years gave higher peak height velocity. Girls with higher animal protein intake at 6–8 years had earlier peak growth. Later age at menarche was associated with lower age at peak growth (*r =* 0.81, *p*<0.05) and lower peak growth velocity (*r*=-0.41, *p*<0.05).

Günther et al. (54), graded A, used data from the DONALD study in Germany to examine the association between dietary protein intake in early- and mid-childhood and timing of puberty; the ages of take-off of the pubertal growth spurt (ATO), age at peak height velocity (APHV), and menarche in girls/voice break in boys. Dietary assessment method is described in Günther et al. (35) BMI, growth, body composition, and sIGF-I above.

Higher animal protein intake (E%) at 5–6 years was associated with earlier puberty. Similar tendency was seen at 3–4 years. Vegetable protein intake was associated with a later ATO. Children with higher animal protein intake at 3–4 and 5–6 years had earlier APHV, while those with high vegetable protein intake had later APHV. Higher animal protein intake (especially milk) at 3–4 years tended (*p*=0.06) to be, and at 5–6 years was (*p*=0.02), associated with earlier menarche/voice break and later for high vegetable protein intake. Adjustment for confounders did not change all of these associations.

The authors conclude that whereas higher animal protein intake at 5–6 years might be related to an earlier ATO, APHV and menarche/voice break, higher intakes of vegetable protein at 3–4 and 5–6 years were associated with delayed puberty.

Remer et al. (55), graded B, investigated as part of the DONALD-study the associations between adrenal androgen (AA) secretion with early and late pubertal markers in a prospective study of 109 Caucasian children. Urine samples were used to measure urinary steroid profile, which was used to determine total AA secretion. Energy and animal protein intake was estimated from 3-day weighed dietary records 1 and 2 years before puberty onset.

AA predicted earlier ages at Tanner stage 2 for pubic hair and breast and genital development, but this was independent of protein intake. Intake of animal protein was independently negatively associated with the ATO and with the APHV and negative association of borderline significance was seen between animal protein intake and age at menarche in girls and voice break in boys. Therefore, the authors concluded that animal protein intake might be involved in earlier pubertal growth.

A paper by Shi et al. (56), graded B, was also based on the DONALD-study and included 137 healthy pre-pubertal 3–12-year-old children. The aim was to investigate if adrenal androgen (AA) production in childhood is associated with body composition and dietary intakes. Diet was, as in the prior study, tested by 3-days’ dietary registration and urine samples were used to measure urinary steroid profile, which was used to determine total AA secretion. Fat mass (FM) predicted most of the variation in AA (5%, *p*<0.0001) and total protein intake to some extent (1%, *p*<0.05). The authors concluded that body fat may relevantly influence the prepubertal adrenarchal androgen status and animal protein may also make a contribution.

#### Complementary search

Rogers et al. (57) graded B, investigated as part of the ALSPAC study, associations between diet at three time points in childhood (3, 7 and 10 years) and age at menarche (AAM). AAM was categorized as before or after 12 years and 8 months, a point close to the median age in this cohort of British girls. Diet was assessed by FFQ at 3 and 7 years and by a 3-days’ un-weighed food diary at 10 years.

Total and animal protein intakes at 3 and 7 years were positively associated with AAM ≤12 years and 8 months. Meat intake at 3 and 7 years was also strongly positively associated with reaching menarche by 12 years and 8 months. The authors concluded that their data suggest that higher intakes of protein and meat in early to mid-childhood may lead to earlier menarche. Note: energy intake at 3 and 7 years is not stated and thus is not possible to deem as credible or not.

#### Conclusion

Three studies, one American study from the 1930–40s and two from the German DONALD-study found an association between increased intake of animal protein in early childhood (around 5–6 years) and earlier puberty. The third paper from the DONALD-study looked at prepubertal hormone levels and concluded that body fat and animal protein intake may increase the hormone levels.

Based on the fact that three of the papers of the original search came from the same group, we first concluded that evidence was slightly too weak to grade it as probable that increased intake of animal protein in childhood is related to earlier puberty, but as the paper found in the complementary search support this conclusion, we judge that the evidence grade increases to probable (grade 2).

### Glucose–insulin metabolism

Table 6 shows a summary of two randomized control studies (both graded B) looking at glucose–insulin metabolism (details are provided in appendix 6).

**Table 6:** Protein intake and outcome glucose-insulin metabolism (2 clinical trials)

Author, year (ref no.) Country Study design (study name if applicable)	No. of participants	Exposure (incl age)	Outcome (incl age)	Effect/association	Study quality comments
Hoppe, 2009, (27)Denmark CT	831 invited, 89 agreed to participate.	2×2 factorial design: 540 ml milk-based drinks, either: 1) whey with low mineral content (Ca and P) (Whey-low), 2) whey with high mineral content (Whey-high), 3) casein with low mineral content (Case-low), 4) casein with high mineral content (Case-high) RQ: To examine the effects of the two major n milk protein fractions, whey and casein, and milk minerals (Ca and P) in a 2×2 factorial design on IGFs and glucose-insulin metabolism	Serum IGF-1, IGFBP, fasting insulin, C-peptide, index of insulin resistance, glucose	No interactions between milk mineral groups (high, low) and milk protein groups (whey, casein). The milk protein intervention groups were combined. Average daily protein intake was increased by 17% by the whey drink, from 58 g/day (2.23 g/kg per day, 12.98 PE%) to 68 g/day (2.56 g/kg per day, 15.42 PE%) (*p*<0.001), and by 51% by the casein drink, from 68 g per day (2.30 g/kg per day, 14.30 PE%) to 103 g per day (3.44 g/kg per day, 23.40 PE%) (*p*<0.001). In the whey group, fasting insulin increased by 21% (*p*=0.006), with no change in IGF-1 (*p*=0.27). In the casein group, serum IGF-1 increased by 15% (*p*<0.0001), whereas there was no change in fasting insulin (*p*=0.36). No independent effects of a high milk mineral intake on IGF-1 and insulin. Increase in serum urea nitrogen (SUN), and the molar ratio of IGF-1/IGFBP-3 was significantly higher in the combined casein-group than in the combined whey group. Conversely, whey increased fasting insulin more than did casein.	B 36% drop-out. No details given. Remaining diet unclear. Energy intake at baseline reported and credible level. Measurement errors not considered Can′t find that they say very much about compliance. They state in Discussion that ‘However, the diet was appropriately recorded, and this has been controlled for in the analysis.’ Intake of energy, protein and milk,+SUN (biomarker for protein intake) was controlled for in the analysis (but how?). Nothing more is said. 2e) They use SUN as a biomarker for protein intake, but don′t say anything about the rest of the dietary intake.
Sandström, 2008 (31) Sweden CT Partly RCT	80 (Healthy GA:36–42 weeks BWT: 2500–5000 g)	Standard vs. two formulas varying in G Lycomacropeptide (GMP) and α-lactalbumin i.e. 3 formulas w. bovine whey fractions rich in α-lactalbumin w. varying GMP vs. breast feeding (as control) All formulas: 1,96 g prot/ 100 kcal.	–Growth –General health –Plasma leptin, insulin, urea nitrogen, amino acids	Formula intake was similar in different groups. Weight gain in the alpha-lactalbumin-enriched formula groups was similar to that of the breastfed infants. The standard formula group gained significantly more weight than did the breastfed infants.All formula-fed infants had significantly higher plasma concentrations of most essential amino acids at 4 and 6 months than did the breastfed infants, and serum urea nitrogen was also higher in the formula-fed infants. Insulin and leptin concentrations did not differ between groups.	B No power calculation reported, compliance unclear, energy intake unclear, results not analysed blind, unclear about between-measurments errors

#### Clinical trials

Hoppe et al. (27) graded B, examined in a double-blinded randomized trial the effects of the two major milk fractions, whey and casein, and milk minerals (Ca and P) on sIGF-I and glucose–insulin metabolism (details about the methodology BMI, growth, body composition, and sIGF-I). Average daily protein intake was increased by 17% by the whey drink and by 51% by the casein drink. In the whey group, fasting insulin increased by 21%. No independent effects of a high milk mineral intake on insulin were found.

Increase in SUN was significantly higher in the casein-group than in the whey group. Conversely, whey increased fasting insulin more than did casein. A limitation of the study, also mentioned by the authors, is that the subjects were allowed to eat their habitual diet, so there might be other factors in the diet contributing to the findings. However, the authors point out that this has been controlled for in the analyses. The results were not changed markedly after controlling for energy intake, protein intake, SUN or milk intake. The authors concluded that whey protein stimulates fasting insulin.

Sandström et al. (31) graded B, did a randomized control study on the effects of exchanging part of the protein in milk-based formulas with α-lactalbumin on insulin levels (and other outcomes). All formulas contained 1.96 g protein/100 kcal, but with differences in the protein composition.

Three different formulas were used; 1) whey-predominant standard infant formula (11% α-lactalbumin, 14% glycomacropeptide [GMP]); 2) α-lactalbumin -enriched formula (25% α-lactalbumin), with GMP accounting for 15% of the protein content and 3) α-lactalbumin-enriched formula (25% α-lactalbumin), with GMP accounting for 10% of the protein content. Breastfed infants were the controls. Measurement errors in the dietary recordings were not considered.

All formula-fed infants had significantly higher plasma concentrations of most essential amino acids at 4 and 6 months than did the breastfed infants, and SUN was also higher in the formula-fed infants. Insulin and leptin concentrations did not differ between groups.

#### Conclusion

Based on the low number of studies, we conclude that evidence is limited-inconclusive (grade 4) that milk protein fractions are related to glucose–insulin metabolism in infancy and childhood.

### Blood pressure

Table 7 shows a summary of studies with outcome blood pressure (details are provided in appendix 7). One paper, a cross-sectional study graded B, was chosen in the systematic review process to be evaluated for the evidence of an association between protein intake and blood pressure.

**Table 7:** Summary table. Protein intake and blood pressure (1 cross-sectional study)

Author, year (ref no.) Country Study design (study name if applicable)	No. of participants	Exposure (incl age)	Outcome (incl age)	Effect/association	Study quality comments
Ulbak, 2004 (58) Denmark Cross sectional on 2.5 year olds, a part of a randomized trial	73	Diet, size	Blood pressure	1-SD increase in protein intake corresponded to diminishing 3 mmHg in systolic blood pressure	B Cross sectional in nature, but it is a study from an intervention among lactating women. power calculations missing, no physical activity (but that might be NA because of young age), information on total energy intake is not found in the article.

#### Cross sectional study

Ulbak et al. (58) graded B, investigated in a cross sectional part of a randomized trial if macronutrients affected blood pressure in 2.5-year-old Danish children. The randomized trial concerned intake of n-3 long chain fatty acids of the mothers while lactating. Mothers kept 7-days food registration when the children were 2.5 years, the age when blood pressure was measured. Measurement errors in the dietary recordings were not considered. E% protein was significantly associated with both systolic and diastolic blood pressure, and g protein/day also significantly associated negatively with systolic blood pressure. The authors concluded that higher protein intake at the age of 2.5 years is associated with lower blood pressure.

#### Conclusion

Based on the low number of studies, we conclude that evidence is limited-inconclusive (grade 4) that higher protein intake is associated with decreased systolic blood pressure in young children.

### Neurodevelopment

Table 8 shows a summary of studies with outcome neurodevelopment (details are provided in appendix 8). Two papers, both prospective cohort studies graded B, were chosen in the systematic review process to be evaluated for the evidence of an association between protein intake and neurodevelopment.

**Table 8:** Protein intake and neurodevelopment (2 cohort studies)

Author, year (ref no.) Country Study design (study name if applicable)	No. of participants	Exposure (incl age)	Outcome (incl age)	Effect/association	Study quality Comments
Morgan, 2004 (37) UK Prospective cohort	144	1. Total red and white meat intake (g) from 4 to 12 months as a continuous variable, i.e. total meat intake over 21 days between 4 and 12 months. 2. Total red and white meat intake (g) from 4 to 16 months as a continuous variable, i.e. total meat intake over 28 days between 4 and 16 months. 3. Total red and white meat intake (g) from 4 to 24 months as a continuous variable, i.e. total meat intake over 42 days between 4 and 24 months.	Neurodevelopment was determined from the mental and motor scales of the Bayley Scales of Infant Development II at 22 months	Meat intake from 4 to 12 and 4 to 16 months was positively and significantly related to psychomotor developmental indices (*p*, 0.02 and 0.013, respectively) but there was no association between breastfeeding and psychomotor developmental indices nor any interaction between meat intake and breastfeeding. Conversely, breastfeeding was positively and significantly related to mental developmental indices (*p*=0.01) but there was no association between meat intake and mental developmental indices or any interaction between breastfeeding and meat intake. These findings remained after adjustment for potential confounding factors.	B Food consumption database not reported, statistical power not reported.
Rask-Nissilä, 2002, (59) Finland Prospective cohort	496	(Energy [kcal]; fat [E%]; saturated, monounsaturated, and polyunsaturated fatty acids [E%]; protein [E%]; and cholesterol [mg/day]) and serum cholesterol concentrations	The neurologic development (speech and language skills, gross motor performance and perception)	High protein intake at 5 and 4 years predicted speech and language skills at 5 years of age.	B Criteria for inclusion/exclusion not clear, loss to follow-up > 50%, associations between dietary exposures not reported, energy adjustment not clear, confounders?

#### Prospective cohort studies

Morgan et al. (37) graded B, studied health outcomes from complementary foods, especially growth and neurocognitive outcome from meat consumption and breastfeeding. Neurocognitive outcome was determined from the mental and psychomotor scales of the Bayley Scales of Infant Development II at the age of 22 months. Red and white meat consumption was calculated from 7-days’ weighed food intake diaries kept regularly at 4-month intervals from 4 months of age to 24 months. Measurement errors in the dietary recordings were not considered.

Total meat intake from all registrations at 4–12 months, that is, three times the 7-days’ registration (*p*<0.02) as well as 4–16 months (four registrations) (*p*<0.013) were positively and significantly related to psychomotor developmental indices. The authors concluded that meat intake from 4 to 16 months was associated with psychomotor development (and increased weight gain) possibly via its protein content. (Note: can the effect of ‘larger child eating more’ be excluded? Larger children, in this population from birth weight 2500 g, are likely to be more developed.)

Rask-Nissilä et al. (59) graded B, tested in 496 children, participating in the prospective STRIP project (the Special Turku Coronary Risk Factor Intervention Study), if dietary and other factors were associated with neurodevelopment. Neurodevelopment was tested at the age of 5 years by using a collection of developmental screening tests. Dietary intake was tested at 8, 13, and 18 months of age and thereafter twice a year. Measurement errors in the dietary recordings were not considered. In boys, stepwise logistic regression showed that protein intake predicted by positive association the outcome in speech and language skill tests at the age of 5 years.

#### Conclusion

Based on the low number of studies, we conclude that evidence is limited-inconclusive (grade 4**)** that higher protein intake is associated with improved neurodevelopment in children.

## Discussion

The overall aim of this SLR was to evaluate recent scientific data on the short- and long-term health effects of different levels of protein intake in infancy and childhood, in order to appraise the current Nordic recommendations, NNR4 (25).

Research questions were developed involving six main outcomes and studies related to these have been presented in this review. A summary of the grading of the evidence for the various outcomes is presented in Table 9. It should be emphasized that the grading of evidence is only based on studies published between January 2000 and December 2011.

**Table 9:** Grading of evidence for health effects associated with protein intake in infancy and childhood in industrialized countries based on an SLR including 34 papers graded A or B and four additional papers graded B from the complementary search

Outcome	Evidence grading	Reference number^1^ (quality grading)
BMI/growth	Convincing evidence **(grade 1)** that higher protein intake in infancy and early childhood is associated with increased growth and/or higher BMI in childhood.	3 (B)^2^, ^3^ 4 (B)^2^ **5 (B)** **4** *13 (B)* *2*, *5* **29 (A)** **2** **30 (B)** **4** 34 (A)^2^ 35 (A)^2^, ^5^ 36 (B)^2^ 37 (B)^2^, ^5^ 38 (B)^2^ 39 (B)^2^ *42 (B)* *2* *44 (B)* *2* 45 (B)^4^, ^6^
	Limited-suggestive evidence **(grade 3)** that intake of animal protein, especially from dairy, have a stronger association with growth than vegetable protein has. The association found between higher intake of milk and increased levels of IGF-I strengthens this	*13 (B)* *2*, *5* **31 (B)** **2**, **5** 35 (A)^2^, ^5^ 37 (B)^2^, ^5^
	Limited-inconclusive evidence (grade 4) that higher protein intake in later childhood is associated with higher BMI in childhood	4 (B)^2^ 39 (B)^2^ 40 (B)^3^, ^4^ *42 (B)* *2* *43 (B)* *3* *44 (B)* *2*
Body composition	Limited-inconclusive evidence (**grade 4**) that protein intake is related to timing of adiposity rebound (AR).	32 (B)^4^ 33 (B)^3^, ^4^
	Limited-inconclusive evidence **(grade 4)** that there is an association between higher protein intake in early childhood and later body fat increases (due to the two A-graded studies not being independent and studies from different groups finding opposing associations).	34 (A)^2^ 35 (A)^2^ 40 (B)^3^, ^5^, ^7^ 41 (B)^3^, ^5^ *42 (B)* *2*, *4*
sIGF-I	Limited-suggestive evidence **(grade 3)** that intake of animal protein, especially from dairy, have a stronger association with growth than vegetable protein has. The association found between higher intake of milk and increased levels of IGF-I strengthens this.	*13 (B)* *2* *14 (B)* *7* **27 (B)** **2** **28 (B)** **2** **30 (B)** **2** 45 (B)^2^, ^6^
Bone	Limited-suggestive evidence **(grade 3)** for a positive association between total protein intake and bone mineral content (BMC) and/or other bone variables in childhood and adolescence.	*14 (B)* *5* **46 (B)** **5** 47 (B)^2^ 48 (B)^2^, ^3^ *49 (B)* *2*, *5* *50 (B)* *2* 51 (B)^4^, ^6^ 52 (B)^6^, ^7^
Puberty	Probable evidence **(grade 2)** that increased intake of animal protein in childhood is related to earlier puberty (final decision reached after the current systematic literature review was supported by a recent paper found by the complementary search.)	53 (B)^2^, ^5^ 54 (A)^2^, ^5^ 55 (B)^2^, ^5^ 56 (B)^2^, ^5^ 57 (B)^2^, ^5^, ^6^
Glucose-insulin metabolism	Limited-inconclusive evidence **(grade 4)** that milk protein fractions are related to glucose-insulin metabolism in infancy and childhood.	**27 (B)** 2, 5 **31 (B)** 2, 5
Blood pressure	Limited-inconclusive evidence **(grade 4)** that protein intake is associated with blood pressure in small children.	*58 (B)* *7*
Neuro-development	Limited-inconclusive evidence **(grade 4)** that protein intake is associated with neurodevelopment in children	37 (B)^2^, ^5^ 59 (B)^2^

1 Bold font indicates clinical trials; plain text indicates cohort studies; italics indicate cross-sectional studies.

2 Positive associations found with protein intake

3 Gender seem to affect the associations

4 No significant associations found with protein intake

5 Associations depending on protein source or amino acid studied

6 From the complementary search (i.e. not to be found in summary tables or appendices)

7 Negative associations found with protein intake

We found the evidence convincing (grade 1) that higher protein intake in infancy and early childhood is associated with increased growth and higher BMI in childhood. Which age period is most sensitive to high protein intake is not clear, but with regard to available data the first 2 years of life seems probable. Due to a scarcity of strong studies there is also limited-inconclusive evidence (grade 4) that protein intake in later childhood is associated with later BMI. There is limited-suggestive evidence (grade 3) that intake of animal protein, especially from dairy, have a stronger positive association with growth than vegetable protein has. The association found between higher intake of milk and increased levels of sIGF-I strengthens this.

There is limited-inconclusive evidence (grade 4) that protein intake is related to timing of AR. The evidence is also limited-inconclusive (grade 4) (due to the two A-graded studies not being independent) that there is an association between higher protein intake in early childhood and later body fat increases. There might also be different effects depending on BMI, phenotypes and gender.

We conclude that evidence is limited but suggestive (grade 3) for a positive association between total protein intake and BMC and/or other bone variables in childhood and adolescence.

Based on the fact that three of the papers of the original search came from the same group, we first concluded that evidence was slightly too weak to grade it as probable that increased intake of animal protein in childhood is related to earlier puberty, but as the paper found in the complementary search support this conclusion, we judge that the evidence grade increases to probable (grade 2).

Regarding other outcomes, this SLR considers that the number of published studies is far too few to enable any conclusions and more research is needed. Evidence is limited-inconclusive (grade 4) that milk protein fractions are related to glucose–insulin metabolism in infancy and childhood. The same level of evidence (grade 4) was found for an association between higher protein intake and decreased blood pressure, as well as for improved neurodevelopment in children.

### Protein intake in infancy and young childhood

Food and nutrient intake during the complementary feeding period, that is, the transition period from milk feeding (breast milk and/or formula) to family foods and its importance for later health has been discussed but not so much studied. Advice given to parents has been and still is more based on custom than scientific evidence.

One major concern during the last decades has been the increasing prevalence of overweight and obesity among both children and adults around the globe. High protein intake during early childhood has been discussed as a possible contributing factor, and Agostoni et al. (60) suggest an increased risk for early AR and later overweight if more than 14 E% comes from proteins in the 6–24 months period.

The protein intake in the RCT-study by Koletzko et al. (29) corresponded at 12 months to 14.0 E% in the low-protein formula group and 16.7 E% in the high-protein formula group (calculated from fig. 2 in the paper), with the higher intake in the latter group associated with increased risk for overweight at 24 months. Scaglione et al. (38) found that 5 year old overweight children had a higher percentage intake of proteins at the age of 1 year than non-overweight children (22 E vs. 20 E%).

To provide advice to health care providers and regulatory bodies, the European Society for Paediatric Gastroenterology, Hepatology and Nutrition (ESPGHAN) published in 2008 a comment on complementary feeding where they stated that *Despite theoretical concerns about the potential effects of different aspects of complementary feeding on later obesity risk, the available evidence is not persuasive* (61). However, several papers of good quality have been published since then and the present SLR supports the conclusion in the paper by Agostoni et al (60).

It can be seen from the studies published on protein intake in the latter half of the first year that there is increased growth and increased risk of overweight later in childhood when the E% from protein at 12 months of age is between 15 and 20 E%. We therefore suggest a mean intake of 15 E% as upper limit at 12 months as there is no risk of too low protein intake at this level but might be increased risk of later overweight with higher intake. This level (15 E%) would also be comparable to the protein content of an average diet among children in the Nordic countries during the first few years (Table 1).

The present SLR focuses on the protein intake in young childhood. The literature is so very scarce on protein intake in older children and adulthood vs. BMI development that it could not be treated in an SLR. Results from some studies on weight loss programs for adults have suggested that a high protein intake can result in more weight loss, but this question is not treated in this SLR.

### Milk and protein intake in a Nordic setting

The intake of protein among children in the Nordic countries is 2–3 times higher than physiological requirements (Table 1). One source of protein is milk and milk products, which are often consumed in large amounts in the Nordic countries. The latest Swedish national study (23) on intake among 4-, 7- and 11-year olds show that at least 25% of all age groups consume more than 500 ml of milk, fermented milk and yoghurt per day, and 5% of boys aged 7 and 11 years consume at least 1 L/day. The reported intake of meat was also large with a median intake of 63, 98 and 98 g/day in the respective age groups. Five percent of 4-year olds reported a meat intake over 150 g/day, and 5% of 7- and 11- year olds reported eating more than 200 g/day. With regard to the results of the present SLR, it seems prudent to advise against too high an intake of protein rich foods, for example, cow's milk, during the first years of childhood.

### Methodological concerns

A difficulty with studies on the effect of protein intake is to ascertain whether any effects are mainly due to protein as such, or to other properties of the protein source (e.g. in dairy products the effects could be due to specific amino acid patterns, peptides, growth factors, minerals or a combination of these). Many studies included in the present SLR do not make this differentiation.

There are conflicting results about the relationship between milk and/or dairy intake and adiposity and body composition in children; some studies show a protective effect while others show negative or no effects (62). There are several possible reasons for this, including differences in methodology both with regard to anthropometric measurements and dietary intake. With regard to the results of the present SLR, we would suggest that it is also important to adjust for the protein intake during the first 1–2 years.

Another important factor is whether individuals with implausible dietary recordings are included or not. Well-performed prospective studies with well-defined and validated dietary assessment methods are needed in all studies aiming to evaluate associations between dietary intake and health outcomes. Many studies included in the present SLR have not considered the validity of the reported dietary intake, which could be problematic. However, protein intake seems to be less affected by faulty reporting than many other nutrients and it is very unlikely that this would cause the associations between reported protein intake and health outcomes found in the present SLR to be inaccurate.

Nordic collaboration with data from prospective longitudinal infant cohorts would be valuable with good possibilities of methodologically strong studies.

## Conclusions

A high intake of protein in infancy and young childhood thus seems to be less than optimal, and associated with increased risk of obesity later in life. The intake of protein in the Nordic countries is, as in many industrialized countries, more than sufficient to meet physiological requirements among children and adults. However, the upper level of a healthy intake is yet to be firmly established. In the meantime, we suggest a mean intake of 15 E% as the upper limit at 12 months as there is no risk of too low protein intake at this level but might be increased risk of later overweight with higher intake. One way to decrease protein intake would be to promote breastfeeding throughout the first year of life or as long as it suits the mother and child, and to avoid too high intakes of protein rich foods, for example, cow's milk.
